# Global expression differences and tissue specific expression differences in rice evolution result in two contrasting types of differentially expressed genes

**DOI:** 10.1186/s12864-015-2319-1

**Published:** 2015-12-23

**Authors:** Youko Horiuchi, Yoshiaki Harushima, Hironori Fujisawa, Takako Mochizuki, Masahiro Fujita, Hajime Ohyanagi, Nori Kurata

**Affiliations:** Plant Genetics Laboratory, Genetic Strains Research Center, National Institute of Genetics, Yata 1111, Mishima, Shizuoka 411-8540 Japan; Transdisciplinary Research Integration Center, Research Organization of Information and Systems, Hulic Kamiyacho 2 F, 4-3-13 Toranomon, Minatoku, Tokyo 105-0001 Japan; Department of Mathematical Analysis and Statistical Inference, The Institute of Statistical Mathematics, 10-3 Midori-cho, Tachikawa, Tokyo 190-8562 Japan; SOKENDAI (The Graduate University for Advanced Studies), 1560-35 Kamiyamaguchi, Hayama, Miura District, Kanagawa Prefecture 240-0115 Japan; Present address: Genome Informatics Laboratory, Center for Information Biology, National Institute of Genetics, Yata 1111, Mishima, Shizuoka 411-8540 Japan; Mitsubishi Space Software Co Ltd, Tsukuba Mitsui Building 14 F, 1-6-1 Takezono, Tsukuba, Ibaraki 305-0032 Japan; Present address: Computational Bioscience Research Center, King Abdullah University of Science and Technology, 4700 KAUST, Thuwal, 23955-6900 Kingdom of Saudi Arabia

## Abstract

**Background:**

Since the development of transcriptome analysis systems, many expression evolution studies characterized evolutionary forces acting on gene expression, without explicit discrimination between global expression differences and tissue specific expression differences. However, different types of gene expression alteration should have different effects on an organism, the evolutionary forces that act on them might be different, and different types of genes might show different types of differential expression between species. To confirm this, we studied differentially expressed (DE) genes among closely related groups that have extensive gene expression atlases, and clarified characteristics of different types of DE genes including the identification of regulating loci for differential expression using expression quantitative loci (eQTL) analysis data.

**Results:**

We detected differentially expressed (DE) genes between rice subspecies in five homologous tissues that were verified using *japonica* and *indica* transcriptome atlases in public databases. Using the transcriptome atlases, we classified DE genes into two types, global DE genes and changed-tissues DE genes. Global type DE genes were not expressed in any tissues in the atlas of one subspecies, however changed-tissues type DE genes were expressed in both subspecies with different tissue specificity. For the five tissues in the two *japonica-indica* combinations, 4.6 ± 0.8 and 5.9 ± 1.5 % of highly expressed genes were global and changed-tissues DE genes, respectively. Changed-tissues DE genes varied in number between tissues, increasing linearly with the abundance of tissue specifically expressed genes in the tissue. Molecular evolution of global DE genes was rapid, unlike that of changed-tissues DE genes. Based on gene ontology, global and changed-tissues DE genes were different, having no common GO terms. Expression differences of most global DE genes were regulated by *cis*-eQTLs. Expression evolution of changed-tissues DE genes was rapid in tissue specifically expressed genes and those rapidly evolved changed-tissues DE genes were regulated not by *cis*-eQTLs, but by complicated *trans*-eQTLs.

**Conclusions:**

Global DE genes and changed-tissues DE genes had contrasting characteristics. The two contrasting types of DE genes provide possible explanations for the previous controversial conclusions about the relationships between molecular evolution and expression evolution of genes in different species, and the relationship between expression breadth and expression conservation in evolution.

**Electronic supplementary material:**

The online version of this article (doi:10.1186/s12864-015-2319-1) contains supplementary material, which is available to authorized users.

## Background

Evolution is brought about by variations both in coding sequences and in gene expression. Many gene sequence comparison studies have been pursued from since the acquisition of various gene sequences from many organisms. One of the important results from these studies is that many homologous proteins have highly conserved functions among species. Thus, many researchers emphasize the importance of gene expression differences caused by changes in *cis*-regulatory elements, leading to divergence in morphology, physiology and behavior [1–4]. Studies of expression evolution were started after the recent development of large-scale transcriptome analysis systems. The first comparative transcriptome study was done using a microarray [5], and many microarray expression evolution studies have since been performed (reviewed in [6–8]). Recent expression evolution studies have been performed with RNA sequencing (RNA-seq) using high-throughout next generation sequencers (reviewed in [9, 10]). Most of these studies (both microarray and RNA-seq) have focused on characterization of the evolutionary forces on gene expression by comparison of expression level variation within species and among species, and evaluate the relative influences of neutral drift, stabilizing selection, directional selection, and balancing selection on gene expression evolution. However, evaluation of the differential expression of a gene is not simple, because the gene expression level is different in different types of cells, and each gene is expressed in a different range of tissues. Expression of some genes could be altered in many tissues in a different species, and expression of some genes could be tissue specifically altered in different species. Since different types of gene expression alteration would result in different effects on an organism, the evolutionary forces that act on them might be different, and different types of genes might show different types of differential expression between species. To confirm this, we study differentially expressed (DE) genes among closely related groups that have extensive gene expression atlases, and clarify the characteristics of different types of DE genes and identify the loci regulating the differential expression by using expression quantitative loci (eQTL) analysis data.

In this study, we characterized DE genes among closely related groups of Asian cultivated rice, *Oryza sativa* L, using publically available sequence and transcriptome data. Rice is an important plant not only as a staple food but also as a model plant, and many valuable data have been accumulated in public databases. Rice has two subspecies, *japonica* and *indica*, and their differentiation has been estimated to have occurred 0.4-0.2 mya [11–13]. A high quality whole genome sequence of a *japonica* variety Nipponbare was determined by BAC-by-BAC sequencing [14], and a whole genome sequence of an *indica* variety 93-11 was determined by shotgun sequencing [15]. We previously analyzed a Nipponbare transcriptome atlas consisting of 25 stages/organs using the Affymetrix rice genome array [16], and an *indica* expression atlas of two varieties was also studied using the same platform [17].

Caution on two points should be exercised when *japonica-indica* DE (*ji*DE) genes are identified from public array data sets. One is the issue of nucleotide polymorphism and the other is the identification of homologous tissue data sets between *japonica* and *indica*. We have previously developed a robust method “Simultaneous detection of nucleotide and expression polymorphisms using Affymetrix GeneChip” (SNEP) to estimate differential expression precisely using probes with different affinities to target RNAs [18]. For the identification of homologous tissue data sets from public databases, we used clustering analysis of those data sets using a set of previously identified *japonica-indica* similarly expressed (*ji*SE) genes that were determined from our Nipponbare and 93-11 shoot and panicle transcriptome array data sets [19].

We used transcriptome atlas data for the characterization of *ji*DE genes according to their gene expression profiles, and classified *ji*DE genes into two types, global and changed-tissues *ji*DE genes. Global type DE genes were not significantly expressed in any tissues of the atlas of one subspecies, but were highly expressed in at least one tissue in the other species. Changed-tissues type DE genes were expressed in both subspecies but differentially expressed in some tissues. We investigated the following characteristics of the two types of *ji*DE genes in comparison with *ji*SE genes: the number expressed in each homologous tissue; the level of tissue specificity; their molecular evolution; their chromosomal gene distribution; and the kinds of genes enriched in each group. In addition to the investigation of *ji*DE genes, we also investigated *indica-indica* DE genes, and classified them into global and changed-tissues types as we did for *ji*DE genes. We discussed causal loci for the two types of DE using the results of eQTL analysis [20].

## Results

### Robust estimation of differential expression considering structural differences between *japonica* and *indica* genes

A potential problem with gene-expression measurements in different species using a single-species array is sequence mismatch. Because the probes on the rice genome array were designed for mainly *japonica*, their target sequences can differ from the target RNAs derived from *indica* at many positions. In the presence of such sequence mismatches or gene model differences, relative hybridization intensities will reflect both differences in transcript abundance level and differences in hybridization affinity. For precise measurement of expression divergence between the two subspecies, we recently developed a robust statistical method, SNEP [18], to analyze the hybridization of probes with target RNAs with sequence polymorphisms. SNEP simultaneously estimates differential gene expression and single feature polymorphisms (SFP) caused by nucleotide polymorphisms, gene structural differences (including alternative splicing between two species), and also gene prediction errors, using transcript hybridization data.

We performed a SNEP two-side test to identify SFP probes and *ji*DE genes from transcript hybridization data of Nipponbare and 93-11 shoot and young panicle. Examples of SNEP analysis are shown in Additional file 1: Figure S1. Some nucleotide polymorphisms between probes and transcripts affected signal intensities. SNEP could detect SFPs in both Nipponbare and 93-11 transcripts, and estimate robust intensity differences. Gene expression changes were estimated discarding probes with SFPs from each probe set. We estimated a gene expression value as the average of the median non-SFP probe intensities for the sample replicates for each probe set. Since the number of replicates for some samples was low in the later analysis, we determined *ji*DE genes using three criteria simultaneously: *p*-value (*p* < 0.05), fold change threshold (>2.5 fold), and highly expressed (log_2_-intensity gene expression value > 7) in either *japonica* or *indica.* Hereafter “highly expressed” means the log_2_-intensity gene expression value is higher than seven. About thirteen thousand genes were highly expressed in each tissue of both strains, and we detected 585 and 1074 *ji*DE genes in panicle and shoot, respectively (Table 1). A total of 1286 genes were detected as *ji*DE genes in shoot or panicle and 12,097 genes were similarly and highly expressed *ji*SE genes between the two subspecies in both shoot and young panicle.

**Table 1:** Gene expression analyses between Nipponbare and 93-11

Tissue	Nipponbare		93-11		Nipponbare or 93-11		
	Expressed^a^	*ji*DE^b^	Expressed^a^	*ji*DE^b^	Expressed^a^	*ji*DE^b^	*j* > *i*
Panicle	13,293	479	13,177	181	14,197	556	450
Shoot	13,768	896	13,477	599	14,897	1035	679
Panicle or Shoot	15,074	1141	14,739	744	16,166	1286	957
Common	11,987	721	11,915	467	12,928	831	457

^a^A gene expression value was defined as the average of replicate median of non-SFP probes log_2_-intensities in a set. We defined highly expressed genes as those having an expression value higher than seven

^b^Detected number of *ji*DE genes among the highly expressed genes by SNEP analysis

### Identification of homologous tissue data sets between *japonica*-*indica*

To identify homologous tissue data sets for the study of *ji*DE genes, we performed cluster analysis on a total of 321 array data sets obtained from Gene Expression Omnibus (GEO) database (http://www.ncbi.nlm.nih.gov/geo/) using hybridization data of the 12,097 *ji*SE genes. The 321 data sets consisted of 121 arrays of 44 Nipponbare tissues, and 200 arrays of three *indica* varieties (ten arrays of two 93-11 tissues; 92 arrays of 36 Minghui63 tissues; and 98 arrays of 39 Zhenshan97 tissues) (Additional file 2: Table S1). After quantile normalization of the 321 arrays and excluding Nipponbare – 93-11 shoot or panicle SFP probe data, we performed hierarchical clustering of the 321 array data using the 12,097 *ji*SE genes in both shoot and young panicle. The expression data were properly grouped according to their similar annotated stages irrespective of their variety as shown the dendrogram in Additional file 3: Figure S2. Therefore batch effects in this study were likely to be smaller than biological effects. A total of 28 clusters were produced by cutting the dendrogram at height 0.75. We selected five tissues, “panicle” in cluster 2, “leaf” in cluster 16, “root” in cluster 17, “endosperm” in cluster 18, and “anther” in cluster 20 for *ji*DE gene detection, because the data for the Nipponbare, Minghui63 and Zhenshan97 varieties were grouped together in a single cluster for those tissues (Additional file 2: Table S1 and Additional file 3: Figure S2).

### Identification and characterization of *ji*DE genes

We performed SNEP analysis on the 43,934 unique probe transcripts in the five selected tissues in order to detect *ji*DE genes. We selected *ji*DE and highly expressed genes in both the Nipponbare-Minghui63 (NM) and Nipponbare-Zhenshan97 (NZ) combinations for the five tissues. The numbers of highly expressed genes were not so different among the five tissues, 12,740 ± 443, however, the numbers of detected *ji*DE genes varied among combinations and tissues ranging from 497 to 1294 (Additional file 4: Table S2).

**Fig. 1:**
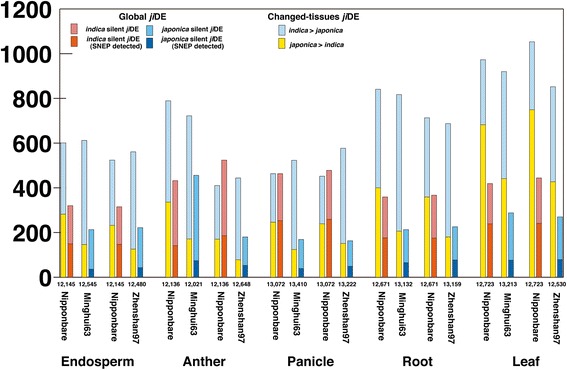
Numbers of highly expressed global and changed-tissues *ji*DE genes in each tissue of a strain. Different types of *ji*DE genes are indicated in the panel by differently colored bars. These numbers are also summarized in Additional file 5: Table S3

To characterize *ji*DE genes detected in the five tissues, we utilized the gene expression atlas from the array data sets of 44 Nipponbare tissues, 36 Minghui63 tissues and 39 Zhenshan97 tissues. We firstly defined silent genes for each strain as global *ji*DE genes. Minghui63 silent genes were highly expressed in a Nipponbare tissue, but their log_2_-intensities did not exceed seven in any 36 Minghui63 tissues. Zhenshan97 silent genes and Nipponbare silent genes identified in a similar manner. Some silent genes had already been detected as *ji*DE genes by SNEP analysis, however, further silent genes were detected by the above tissue expression profiling analysis, and added to the set of *ji*DE genes. On average 7.9 ± 1.8 % of the highly expressed genes in each tissue of the three strains were *ji*DE genes (Additional file 5: Table S3). In the following characterization, *ji*DE genes were classified as global or changed-tissues and all comparisons concerned only highly expressed genes (log_2_-intensity gene expression value > 7 in at least one tissue). We compared the numbers of global and changed-tissues *ji*DE genes in each tissue of the three strains (Fig. 1 and Additional file 5: Table S3). The numbers of *indica* silent genes that were highly expressed in Nipponbare tissues were not so varied by tissue, 412.1 ± 66.1 (Fig. 1 and Additional file 5: Table S3). The expressed numbers of Nipponbare silent genes were also similar across *indica* tissues, 216.0 ± 40.2, except for Minghui63 anther, where 456 Nipponbare silent genes were highly expressed (Fig. 1 and Additional file 5: Table S3). The numbers of Nipponbare silent *ji*DE genes were smaller than those of *indica* silent *ji*DE genes in all cases. The smaller numbers of Nipponbare silent genes compared to *indica* ones could be due to the design of the Affymetrix array, based mainly on *japonica* transcripts. The detected numbers of changed-tissues *ji*DE genes were not so different between *japonica* and *indica* in the same tissues, however, the differences among tissues were large (Fig. 1 and Additional file 5: Table S3). In anther, the detected numbers of changed-tissues *ji*DE genes (*p* < 2.2x10-^16^) and global *ji*DE genes (*p* = 7.9x10^−8^) were different between the Nipponbare-Minghui63 and Nipponbare-Zhenshan97 combinations (Fig. 1 and Additional file 5: Table S3).

### Tissue specificity of *ji*DE genes

**Fig. 2:**
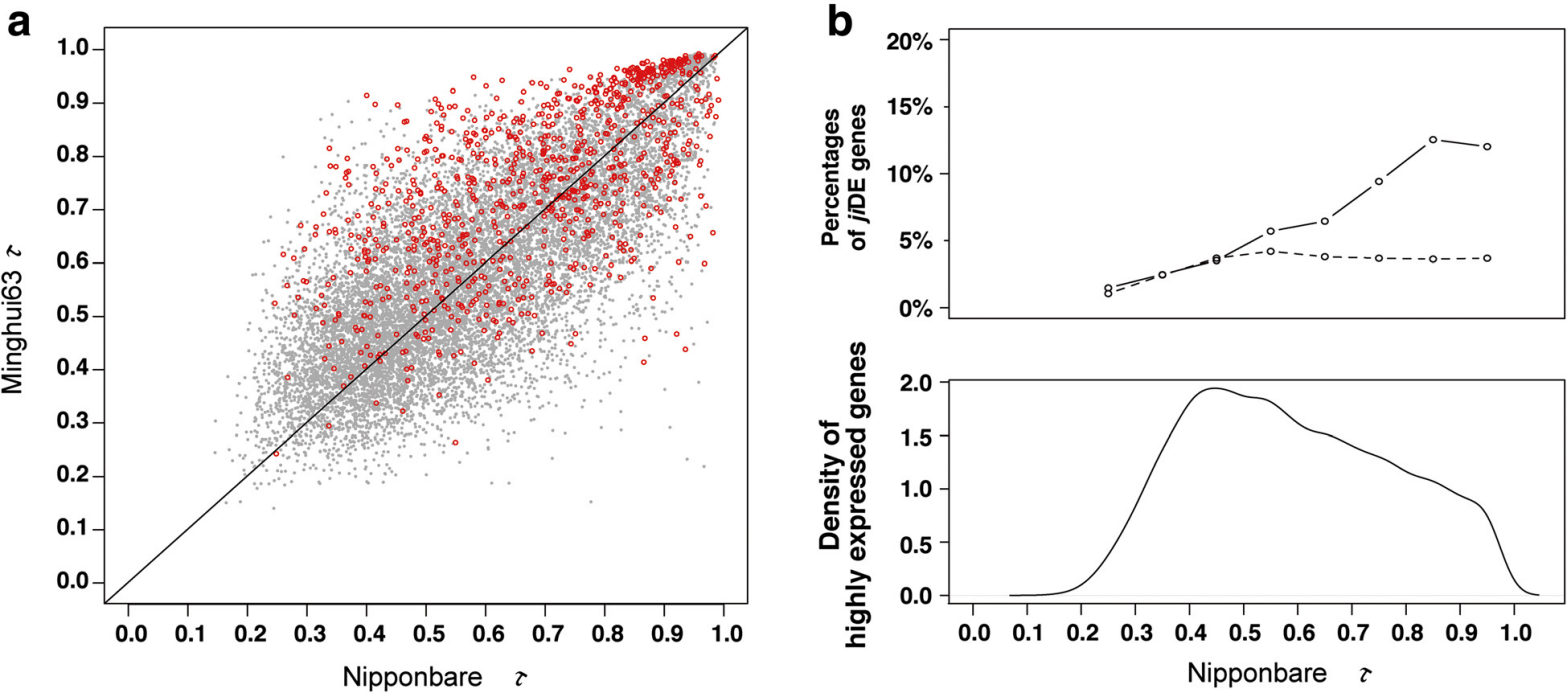
Distribution of tissue specificity index, *τ*, of highly expressed genes in anther. Yanai’s tissue specificity index, *τ,* is 1 minus an average ratio of non-maximal tissue expression intensities to the maximal tissue expression intensity [23]. When *τ* is 1, the gene is expressed in only one tissue. When *τ* is 0, the gene expression intensities of all tissues are the same. **a:** scatter plots of Nipponbare *τ* and Minghui63 *τ* of highly expressed genes in Nipponbare or Minghui63 anther excluding global *ji*DE genes. Nipponbare *τ* and Minghui63 *τ* were calculated using 44 and 36 tissues, respectively (Additional file 2: Table S1 and Additional file 2: Figure S2). Red circles present changed-tissues *ji*DE genes. **b:** Percentages of changed-tissues (smooth line) and Minghui63 silent (broken line) *ji*DE genes among Nipponbare anther highly expressed genes in a 0.1 *τ* window are shown in the top panel, and a density plot of Nipponbare *τ* for the highly expressed genes in Nipponbare anther is shown in the bottom panel

We analyzed the tissue specificity of the highly expressed genes using the tissue specificity index *τ*, which gives a value between 0 and 1 based on the expression values [23]. If the index for a gene is 0 expression is constant in all tissues, while if the index is 1 expression is specific to a single tissue. We used 44, 36 and 39 tissue data sets for the calculation of Nipponbare *τ*, Minghui63 *τ*, and Zhenshan97 *τ*, respectively. After removing the global *ji*DE genes, Nipponbare *τ* and Minghui63 *τ* of highly expressed genes in Nipponbare and Minghui63 anther were compared by scatter diagram as shown in Fig. 2a. Although only five tissues were common between the 44 Nipponbare and 36 Minghui63 arrays (Additional file 3: Figure S2), the *τ* values were in good agreement with each other including those for changed-tissues *ji*DE genes (Fig. 2a). The *τ* value agreements were also maintained in the other tissue or rice variety combinations (Additional file 6: Figure S3). A density plot of the *τ* values for Nipponbare anther genes is shown in Fig. 2b. Genes with *τ* less than 0.2 were rare. This does not mean that there were no constitutively expressed genes, but most genes had expression differences among tissues including those often considered as “housekeeping genes”. The Nipponbare *τ* values of elongation factor LOC_Os03g08010, ubiquitin LOC_Os06g46770, glyceraldehyde-3-phosphate dehydrogenase LOC_Os08g03290, alpha-tubulin LOC_Os03g51600, and beta-actin LOC_Os03g50890 were 0.164, 0.274, 0.349, 0.466, and 0.683, respectively. The density peak of the *τ* distribution was at 0.45, and the average was 0.6. The percentages of Minghui63 silent and changed-tissues *ji*DE genes in Nipponbare anther were investigated using a sliding window of *τ* of 0.1 (Fig. 2b top panel). The percentage of Minghui63 silent genes did not dependent on *τ*, however, changed-tissues *ji*DE genes were overrepresented in more tissue-specifically expressed genes, being biased towards higher *τ* values. The bias of changed-tissues *ji*DE genes towards higher *τ* values and the lack of a relationship between global *ji*DE genes and *τ* values was also observed in the other tissues in any rice variety combination (Additional file 6: Figure S3). The differences in *τ* preferences between global and changed-tissues *ji*DE genes suggest that the mechanisms causing expression alterations are different between them.

**Fig. 3:**
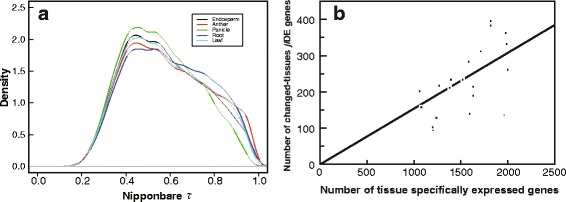
Relationships between tissue specifically expressed genes and changed-tissues *ji*DE genes. Densities of Nipponbare *τ* for highly expressed genes in five Nipponbare tissues: endosperm, anther, panicle, root, and leaf, are plotted in panel **a**. The relationship between the number of changed-tissues *ji*DE genes and the number of tissue specifically expressed genes (Nipponbare *τ >* 0.8)excluding global *ji*DE genes of the five tissues of Nipponbare, Minghui63, and Zhenshan97 is shown in panel **b**. The linear regression line, which passes through the origin of the coordinate, was calculated without a plot () of Nipponbare anther specifically expressed genes and *ji*DE genes to Zhenshan97, and shown in panel B

We compared density plots of *τ* values for highly expressed Nipponbare genes in each tissue and found that the numbers of tissue specifically expressed genes were different across the five tissues (Fig. 3a). To investigate the relationship between the abundances of tissue specifically expressed genes and changed-tissues *ji*DE genes in a tissue, the numbers of genes in with *τ* > 0.8 were plotted as a scatter diagram (Fig. 3b and Additional file 5: Table S3). For the tissue specifically expressed genes, the numbers of changed-tissues *ji*DE genes and those of highly expressed genes showed good linear correlation, which passed through the origin of the coordinate with an inclination angle 0.154 and a coefficient of determination 0.534, except for the plot of Nipponbare anther tissue specifically expressed genes and *ji*DE genes to Zhenshan97. This means that the proportion of changed-tissues *ji*DE genes among tissue specifically expressed genes was similar in many tissues, and three times higher than the average (5.3 ± 1.5 %) among all changed-tissues *ji*DE genes (Additional file 5: Table S3). Therefore differences in the numbers of changed-tissues *ji*DE genes among tissues are considerably dependent on the abundance of tissue specifically expressed genes in each tissue.

To examine whether changed-tissues differential expression generally occurred in the tissue displaying highest expression for each gene in the transcriptome atlas, we counted the numbers of genes that showed the highest expression in the respective transcriptome atlas for the five tissues, (“N atlas highest”, “M atlas highest”, and “Z atlas highest” in Additional file 5: Table S3). These numbers are dependent on the other tissues represented in the atlases. “N atlas highest” of root was the highest among the five tissues of Nipponbare; however, “M atlas highest” and “Z atlas highest” of root were lowest among the five tissues of Minghui63 or Zhenshan97, although about 90 % genes in root had similar expression intensity among them. Our observed gene numbers for the atlas highest could be overestimated, because the expression atlases we used did not cover every tissue throughout the life cycle. The proportion of the atlas highest expressed genes which were tissue specifically expressed changed-tissues *ji*DE genes with *τ* > 0.8 varied from 3.9 % (5/128) of Minghui63 panicle, to 81.7 % (255/312) of Minghui63 anther (Additional file 5: Table S3). There was no observable relationship between occurrence of changed-tissues *ji*DE gene expression and the highest expression tissue of the atlas.

**Fig. 4:**
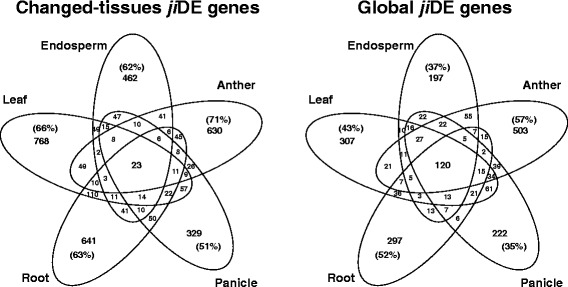
Overlap of *ji*DE genes detected in the five tissues of the NM combination. Percentages of tissue specifically detected *ji*DE genes in each tissue were indicated in parentheses

The different prevalence of *τ* values between global and changed-tissues *ji*DE genes effected the overlap of detected *ji*DE genes in the five tissues. The Venn diagrams in Fig. 4 represent the overlap of 3513 changed-tissues and 2119 global *ji*DE genes detected in the five tissues in the NM combination. Since changed-tissues *ji*DE genes were biased towards higher *τ* values, the percentages of tissue specifically detected changed-tissues *ji*DE genes were higher than those of global *ji*DE genes. On the other hand, since the *τ* distribution of global *ji*DE genes obtained in an active strain was as broad as that of all highly expressed genes, the commonly detected percentage among the five tissues of global *ji*DE genes was more than five times higher than that of changed-tissues *ji*DE genes. These tendencies were also observed in the NZ combination (Additional file 7: Figure S4).

### Molecular evolution of *ji*DE genes

**Fig. 5:**
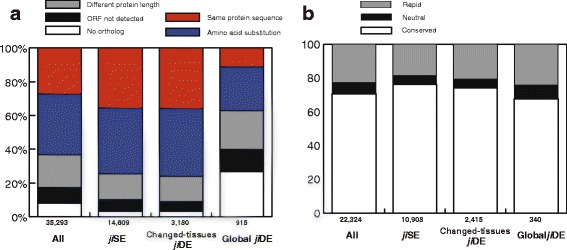
Molecular evolution of Nipponbare highly expressed *ji*DE genes. In panel **a**, the proportions of the five types of orthologous gene pairs between Nipponbare and 93-11 are shown for the four gene categories. The five types of orthologous gene pairs were as follows: 1) No ortholog: no orthologs in the 93-11 genome, 2) ORF not detected: ORF not detected in the 93-11 genome, 3) Different protein length: predicted 93-11 protein length was different, 4) Amino acid substitution: the predicted 93-11 protein was the same length with some amino acid substitutions, and 5) Same protein sequence: the predicted 93-11 protein was the same sequence. The four gene categories were as follows: 1) all genes: ORF were predicted in Nipponbare genome, 2) *ji*SE genes: Nipponbare highly expressed genes in any of endosperm, anther, panicle, root, and leaf, without expression differences in the NM combination, 3) changed-tissues *ji*DE genes: Nipponbare highly expressed *ji*DE genes detected in any of the five tissues in NM combination, and not Minghui63 silent genes, and 4) global *ji*DE genes: highly expressed genes in any of the Nipponbare five tissues, but not expressed in any of the 36 Minghui63 tissues. Numbers of genes in each category are indicated at the bottom of each column. In panel **b**, the proportions of conserved (*K*_a_/*K*_s_ < 0.75), and neutrally (0.75 ≤ *K*_a_/*K*_s_ < 1.25) or rapidly (*K*_a_/*K*_s_ ≥ 1.25) evolved orthologous gene pairs with the same predicted protein length are shown for the four gene categories

We consider *japonica-indica* differential expression of a gene as the result of expression evolution during *japonica-indica* differentiation. We examined relationships between expression evolution and molecular evolution by comparison of molecular evolution among *ji*SE genes, changed-tissues *ji*DE genes, and global *ji*DE genes. We selected 35,293 genes which had open reading frame (ORF) predictions in the TIGR5 [21] or RAP2 [22] annotations from among the 43,932 unique probe transcripts selected earlier. To identify ortholog gene pairs between *japonica* and *indica*, we performed BLAT searches of the 35,293 *japonica* Nipponbare transcript sequences against the 93-11 BGI 2003-08-01 genome sequence [15]. We categorized the Nipponbare transcripts into five types by the results of the *indica* ortholog BLAT search; 1) no orthologs: Nipponbare transcript sequence was not aligned on the 93-11 genome, 2) ORF not detected: an orthologous sequence was detected on the 93-11 genome, but the first methionine was not found, 3) different protein length: insertions deletions or mutations that induced frame shifts leading to premature stop or read through, or disruption of the stop codon, 4) amino acid substitution: predicted *indica* protein length was the same as *japonica* one, but its amino acid sequence was different, and 5) same protein sequence: predicted *indica* protein sequence was identical to *japonica* one (see methods). Comparing only highly expressed genes, the proportions of the five types of *indica* orthologs of Nipponbare were compared among *ji*SE genes, changed-tissues *ji*DE genes, and global *ji*DE genes for the five tissues in the NM combination (Fig. 5a and Additional file 8: Table S4). The proportion of the five ortholog types in changed-tissues *ji*DE genes was similar to that in *ji*SE genes. However, a higher proportion of diverged or disrupted gene structures were found in Nipponbare – Minghui63 global *ji*DE genes. For predicted orthologs with the same amino acid sequence length, the ratios of the number of non-synonymous substitutions per non-synonymous site (*K*_a_) to the number of synonymous substitutions per synonymous site (*K*_s_) were calculated, and according to *K*_a_/*K*_s_, the ortholog gene pairs were classified into three types: conserved; *K*_a_/*K*_s_ < 0.75, neutral; 0.75 ≤ *K*_a_/*K*_s_ < 1.25, and rapid; *K*_a_/*K*_s_ ≥ 1.25. The proportion of the three types in changed-tissues *ji*DE genes was also similar to that in *ji*SE genes, however, the proportion of the rapidly evolved genes in global *ji*DE genes was higher than those in changed-tissues *ji*DE genes and *ji*SE genes (Fig. 5b and Additional file 8: Table S4). This high proportion of rapidly evolved global *ji*DE genes is not caused by positive selection, but results from a lack of selection, because about two thirds of the random mutations in the ORFs of silenced genes induce non-synonymous changes. Since 27 % of global *ji*DE genes had no orthologs in the 93-11 genome and the proportion of in the “Same protein sequence” category among global *ji*DE genes (10.6 %) was lower than in *ji*SE genes (33.9 %) or changed-tissues *ji*DE genes (34.6 %), it was reasonable to consider the possibility that *indica* silent genes expressed but the expressions were not observed due to polymorphisms in their probe targets. We have previously shown [18] that as long as less than half of the probes in a probe set are polymorphic (SFP robes), two-sided SNEP can simultaneously robustly estimate SFPs and gene expression level, and the gene expression levels in *indica* can be determined after the elimination of SFP probes. We estimated the number of genes whose expression level in *indica* 93*-*11 cannot be measured using the Affymetrix rice genome array due to polymorphism using the 26,936 SFPs that we detected by 93-11 genome DNA hybridization [19] (Additional file 8: Table S4). Among the 43,932 genes, the expression levels of only 920 genes (2.1 %) were unmeasurable in *indica*. Although unmeasurable genes were overrepresented in *indica* silent genes (13 %), the expression level of more than 87 % of *indica* silent genes could be determined (Additional file 8: Table S4). We expect the situation to be similar in Minghui63 and Zhenshan97 expression of most of *indica* silent genes is not unmeasurable due to polymorphism, but the genes are genuinely silent in *indica*.

### Distribution of *ji*DE genes on rice genome

**Fig. 6:**
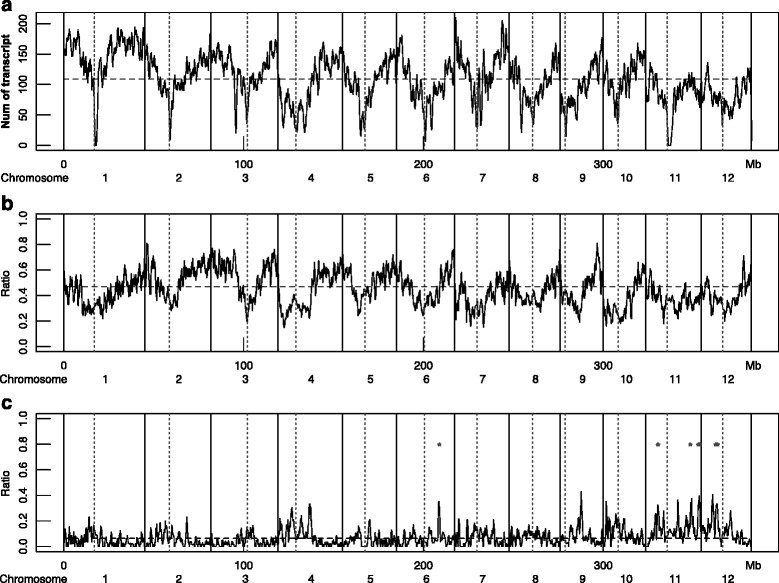
Distribution of genes in Nipponbare genome. The numbers of the mapped 41,750 genes in overlapping every 1 Mb segments with a step width 0.1 Mb on the Nipponbare genome (IRGSP build 4) are shown in panel **a**. The ratio of the highly expressed genes in any of the five tissues per the 100 mapped genes is shown in panel **b**. The ratio of the mapped 2660 Minghui63 silent genes among the 20,779 Nipponbare highly expressed genes are shown in the panel **c**. If the silent *ji*DE gene to highly expressed gene ratio was three times higher than the 3rd quartile value of the ratio distribution and the number of highly expressed genes in the window was more than the first quartile value, 37, of the distribution of highly expressed genes per 100 mapped gene window, we marked red asterisks (*) to indicate silenced genes were exceptionally enriched in that region

To examine whether *ji*DE genes detected in the five tissues of NM combination were uniformly distributed throughout the genome, we mapped four sets of genes on the IRGSP ver.4 Nipponbare genome (http://rgp.dna.affrc.go.jp/E/IRGSP/Build4/build4.html): 1) all 43,932 unique probe transcript genes from the rice genome array; 2) Nipponbare or Minghui63 highly expressed genes in each tissue, 3) changed-tissues *ji*DE genes in each tissue, and 4) Minghui63 silent *ji*DE genes, which expressed in any of the five Nipponbare tissues. We could map 41,748 genes out of the 43,932 unique probe transcript genes on the 382.2 Mb Nipponbare genome by BLAT search. The numbers of the mapped genes in a 1 Mb sliding window with a step width of 0.1 Mb are shown in Fig. 6a. The gene density was low at each centromere region, increasing toward each end. Since gene density was not uniform, the ratio of about 13,000 Nipponbare or Minghui63 highly expressed genes in each tissue to every 100 mapped genes were investigated with a step width of ten mapped genes. The distributions of the highly expressed genes were similar among the five tissues, and there were no enriched regions (Additional file 9: Figure S5 and Additional file 10: Figure S6). The ratios of mapped Nipponbare highly expressed genes in any of the five tissues per 100 mapped genes are shown in Fig. 6b, and the ratios of 1073 mapped Minghui63 silent genes to the 19,567 Nipponbare highly expressed genes are shown in Fig. 6c. We considered *ji*DE genes to be clustered if the *ji*DE ratio in a window was three times higher than the 3rd quartile value of *ji*DE ratios and highly expressed genes were not sparse in the window. The Minghui63 silent genes were not evenly distributed on the genome, and there were five clusters on chromosomes six, 11, and 12. Since there were some *ji*SE genes interspersed between the Minghui63 silent genes within the silent gene cluster regions, the gene silencing in Minghui63 was not uniform throughout the entire cluster regions. For each tissue, the ratio of changed-tissues *ji*DE genes to highly expressed genes among every 100 mapped genes was also surveyed to identify changed-tissues *ji*DE enriched regions. Most changed-tissues *ji*DE genes in the NM combination were evenly distributed among the highly expressed genes, however, there a few clustered changed-tissues *ji*DE genes of endosperm, panicle, and root were found on chromosomes 1, 11, and 1, respectively (Additional file 11: Figure S7). Most changed-tissues *ji*DE genes in the NZ combination were also evenly distributed among the highly expressed genes, with the exceptions of clustered anther changed-tissues *ji*DE genes on chromosomes 9 and 11, panicle changed-tissues *ji*DE genes on chromosomes 4 and 11, and root changed-tissues *ji*DE genes on chromosome 1 (Additional file 12: Figure S8). We could not find any common features of the clustered changed-tissues *ji*DE genes.

### Annotation and gene ontology enrichment analysis of *ji*DE genes

Although rice gene annotations provide limited information, we tried to identify functional trends among *ji*DE genes. We first focused on transposable element-related (TE-related) genes (Additional file 13: Table S5). In the rice genome, there are substantial numbers of TE-related genes. For example, 16,178 of 56,278 TIGR5 annotated genes were TE-related genes (http:rice.plantbiology.msu.edu/). Since many TE-related genes are multiple copy genes and we selected unique probe transcripts for this study (see Methods), the proportion of TE-related genes in our analysis was low, and 1788 out of the 33,824 annotated genes (5.3 %) were annotated as TE-related (Additional file 13: Table S5). The number of expressed TE-related genes in the five tissues was small. Only 347 of the 18,357 annotated genes that were highly expressed in any of the five Nipponbare tissues, were TE-related genes (1.9 %) (Additional file 13: Table S5). However, TE-related genes were significantly over-represented (*p* < 2.2x10^−16^) among Minghui63 silent genes - 5.9 % of the annotated Minghui63 silent genes were TE-related genes (Additional file 13: Table S5). The proportion of TE-related genes among the annotated Nipponbare silent genes that were highly expressed in Minghui63 was 2.9 %, and that expressed in Zhenshan97 was 3.1 % so TE-related gene over-representation in Nipponbare silent genes was not so high but was still obvious (Additional file 13: Table S5).

Gene ontology (GO) provides important information for the characterization of *ji*DE genes, however, only 18,987 out of the 43,932 genes in this study have been assigned GO terms by the Gramene database (http://www.gramene.org/). Using this limited information, we performed GO enrichment analysis to compare global *ji*DE genes, and changed-tissues *ji*DE genes that were detected in the five Nipponbare or Minghui63 tissues. We used Fisher’s exact test to infer enrichment compared with all 18,987 GO assigned genes. The number of GO term assigned NM changed-tissues *ji*DE genes was 2387 out of 3513. Thirty-two GO terms were enriched (*p* < 0.01), and the enriched terms included “response to stress”, “response to cold”, “catalytic activity”, among others. (Additional file 14: Table S6). The number of GO assigned NM global *ji*DE genes was 785 out of 2119. Twenty-one GO terms were enriched, and “apoptosis”, “protein phosphorylation”, and “defense response” were highly enriched (*p* < 1.0E-10) in the “biological process” domain (Additional file 14: Table S6). There were no commonly enriched GO terms between global and changed-tissues DE genes. We compared GO enrichment between Minghui63 silent *ji*DE genes and Nipponbare silent *ji*DE genes, and ten GO terms including “defense response”, “apoptosis”, and “protein phosphorylation” were commonly enriched in both sets of silent genes (Additional file 15: Table S7). In the NZ combination, seven GO terms were also enriched in both sets of silent genes (Additional file 16: Table S8). Therefore similar types of genes related to “defense response” and “protein phosphorylation”, were silenced in both *japonica* and *indica* rice.

Since changed-tissues *ji*DE genes showed tissue dependent expression differences where for the same gene either the *japonica* or *indica* allele could be more highly expressed depending on the tissue; GO enrichment analysis of changed-tissues *ji*DE genes with *japonica* higher expression and those with *indica* higher expression was performed to infer enrichment compared with the highly expressed genes, and compared in each tissue. For each category, between three and 34 GO terms were enriched, and except for in panicle and root, there were no commonly enriched GO terms between *japonica* higher *ji*DE genes and *indica* higher *ji*DE genes in the other tissues (Additional file 17: Table S9 and Additional file 18: Table S10). In panicle specific changed-tissues *ji*DE genes of the NM combination, “response to stress” was commonly enriched, and in root changed-tissues *ji*DE genes, “oxidoreductase activity”, “catalytic activity”, “cation binding”, and “heme binding” were commonly enriched (Additional file 17: Table S9). “Response to high light intensity”, “hydrolase activity, hydrolyzing O-glycosyl compounds”, “catalytic activity”, and “carbohydrate metabolic process” were commonly enriched GO terms in root changed-tissues *ji*DE genes of the NZ combination (Additional file 18: Table S10).

### Expression evolution within *indica*

The characteristics of *ji*DE genes are summarized in Table 2, and these characteristics were the same in both the NM and NZ combinations. However, the numbers of detected *ji*DE genes were different between the two combinations. For global *ji*DE genes among the three strains, their expression patterns in each strain were categorized into two types (Additional file 19: Figure S9 and Additional file 20: Table S11): expressed only in one strain but silent in the other two strains, or silent only in one strain but expressed in the other two strains. Excepting anther, 61.6 ± 1.7 % of global *ji*DE genes were commonly detected in both the NM and NZ combinations, and these gene expressions differed only in Nipponbare (Additional file 20: Table S11). The other 40 % of global *ji*DE gene expressions were uniquely detected in either the NM or NZ combination. The number of uniquely detected global genes in the NM combination was not so different from that in the NZ combination. However, in anther, only 50 % of global *ji*DE genes were expressed similarly between Minghui63 and Zhenshan97; Minghui63 expressed genes and Zhenshan97 silent genes were enriched in anther among global *ji*DE genes (Additional file 19: Figure S9 and Additional file 20: Table S11).

**Table 2:** Characteristics of global and changed-tissues *ji*DE genes

	Global *ji*DE genes.	Changed-tissues *ji*DE genes.
Detected number of *ji*DE genes	•Tissue independent.	•Tissue dependent.
	•More *indica* silent genes were detected than *japonica* silent genes.	•Numbers of highly expressed gene were similar between *japonica* and *indica* strains in each tissue.
Tissue specificity index (*τ*) dependence	•Independent.	•over-represented in higher *τ* genes.
Gene structure	•Largely changed.	•Same as *ji*SE genes.
Molecular evolution	•Free from constraints.	•Same constraints as *ji*SE genes.
Distribution on the Nipponbare genome	•Some genes were clustered.	•Few genes were clustered.
TE related genes	•Enriched.	•Same as *ji*SE genes.
Enriched GO terms.	•No commonly enriched term between global and changed-tissues *ji*DE genes.	
	•“apoptosis”, “protein phosphorylation”, “defense response” etc.	•“response to stress”, “response to cold”, “response to hydrogen peroxide”, and etc.
GO enriched terms between *japonica* higher expressed genes and *indica* higher ones	•Similar terms were enriched between *japonica* and *indica* silent genes.	•Most enriched terms were different between them.

Commonly detected changed-tissues *ji*DE genes between the NM and NZ combinations were investigated in each tissue (Additional file 21: Figure S10. and Additional file 22: Table S12). The ratios of NM-NZ commonly detected changed-tissues *ji*DE genes were lower than those for global *ji*DE genes, and varied from 16.2 to 55.5 % in the five tissues.

A substantial number of changed-tissues *ji*DE genes were detected only in either the NM or the NZ combination, therefore, we performed SNEP analyses between Minghui63 and Zhenshan97 (MZ) combination in the five tissues and identified MZDE genes (differentially expressed between the two *indica* varieties). The detected numbers of MZDE genes were varied widely among tissues, from 109 in endosperm to 651 in anther. Notably, the number of MZDE genes (*indica*-*indica*) in anther was greater than the number of NZ *ji*DE genes (*japonica*-*indica*) (Table 3). It was also noteworthy that there were several three-way DE genes among *ji*DE genes, whose expression levels were different in each of the three strains. In general, modern *indica* cultivars have integrated *japonica* fragments in their genomes [24], thus one possible explanation is that differences in detected *ji*DE genes between the NM and NZ combinations are not due to expression evolution within *indica* varieties but due to differences in integrated *japonica* fragments between Minghui63 and Zhenshan97. However, three-way DE genes would guarantee expression evolution within *indica* varieties. We estimated the expected numbers of three-way DE genes for each tissue, supposing that the proportion of three-way DE genes among the common NM-NZ *ji*DE genes would be the same as that of MZDE genes among the highly expressed genes (Table 3). In fact, the observed numbers of three-way DE genes in the five tissues were higher than the expected ones, and the observed numbers were significantly higher in three out of the five tissues (Table 3). This would suggest that expression evolution within *indica* preferentially occurred in *ji*DE genes. If differences in integrated *japonica* fragments between Minghui63 and Zhenshan97 generated some of the MZDE genes, the expected number of three-way DE genes would increase, and the apparent preferential occurrence of *indica-indica* expression evolution in *ji*DE genes would decrease.

**Table 3:** Changed-tissues DE genes among the three strains

	Endosperm	Anther	Panicle	Root	Leaf
Highly expressed genes^a^	13,533	13,571	14,183	14,024	13,993
Nipponbare-Minghui63 DE genes	730	852	626	989	1134
Nipponbare-Zhenshan97 DE genes	645	516	627	836	1148
Minghui63-Zhenshan97 DE genes	109	651	203	110	464
Common *ji*DE genes^b^	446	191	312	651	670
Observed three-way DE genes^c^	7	10	23	10	33
Expected three-way DE genes^d^	3.6	9.2	4.5	5.1	22.2
*p* value^e^	0.0956	0.733	3.67E-10	0.0382	0.0276

^a^Number of highly expressed genes in any of the three strains excluding silent genes

^b^Number of commonly detected changed-tissues DE genes in the both NM and NZ combinations

^c^Number of observed three-way DE genes, whose expression level was different in each of the three strains

^d^Numbers of expected three-way DE genes were based on the hypothesis that proportion of three-way DE genes among Common *ji*DE genes would be the same as that of Minghui63-Zhenshan97 DE genes among the highly expressed genes

^e^ *p* values of the hypothesis were evaluated by Fisher’s exact test

**Fig. 7:**
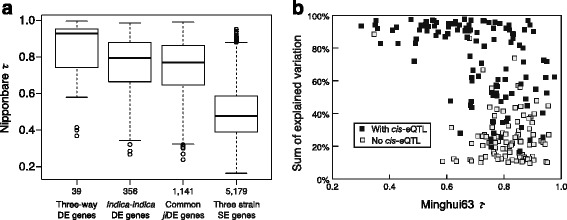
Tissue specificity index (*τ*) and variations explained by detected eQTLs for changed-tissues *indica-indica* DE genes. Box plots of Nipponbare *τ* distributions of the following four different types of genes were shown in panel **a**. “Three-way changed-tissues DE genes”: all expression levels among the three strains were different in one tissue and the expression levels in the three strains were same in the other four tissues. “*Indica-indica* changed-tissues DE genes”: expression levels in Minghui63 and Zhenshan97 were different and the expression level in Nipponbare was the same to either in Minghui63 or in Zhenshan97 in one tissue, and the expression levels in the three strains were same in the other four tissues. “Common changed-tissues *ji*DE genes”: the expression level in Nipponbare was different from the two *indica* strains and the expression level in the two *indica* strains was the same in one tissue, and the expression levels in the three strains were same in the other four tissues. “Three strain SE genes”: expression levels in the three strains were same in the five tissues. Numbers of genes in each category are indicated at the bottom of each box plot. Scatter plots between Minghui63 *τ* and the sum of explained variation by detected eQTLs [20] for changed-tissues MZDE genes at the early seedling stage are shown in panel **b**. The sum of explained variation means a simple summation of explained expression variation across RILs by all detected eQTLs for each gene (Additional file 24: Table S14). Black square genes had *cis*-eQTL among the detected eQTLs. Gray square genes had no *cis*-eQTL among the detected eQTLs

To compare *τ* distributions among three types of changed-tissues DE genes, three-way DE genes, *indica-indica* DE genes, and common *ji*DE genes, we selected genes that showed the respective DE type in only one tissue and SE among the three strains in the other four tissues, since some genes showed different DE types in different tissues. The Nipponbare *τ* medians of the three types of DE genes were higher than that of three strain SE genes. The median *τ* value of three-way DE genes was highest, and that of *indica-indica* DE genes was higher than that of common *ji*DE genes (Fig. 7a). Recent changed-tissues expression evolution might therefore preferentially occur on tissue specifically expressed genes.

### Expression quantitative trait loci (eQTL) analysis of global and changed-tissues DE genes

Wang et al. [20] performed eQTL analysis for TIGR5 transcript units at the early seedling stage using the Affymetrix Rice Genome array and 110 recombinant inbred lines (RILs) from a cross between Minghui63 and Zhenshan97. They also obtained array data for the two parents at the same stage with three biological replicates (GSM560089-94). To investigate whether global and changed-tissues DE genes were regulated by *cis*- or *trans*-eQTLs, we investigated global and changed-tissues MZDE genes at the early seedling stage using the two parents array data by SNEP analysis and examination of their tissue expression profiles as described above. We detected 95 global and 273 changed-tissues MZDE genes at this stage, and of them 68 and 250 were annotated TIGR loci respectively (Table 4). Wang et al. [20] detected eQTLs for 57 global and 211 changed-tissues MZDE genes, and the eQTL detection ratios were similar between global and changed-tissues MZDE genes (Additional file 23: Table S13 and Additional file 24: Table S14). The detected eQTLs for global MZDE genes well explained expression variations across RILs, 86 % on average. Conversely, for changed-tissues MZDE genes, the sum of variation explained by the detected eQTLs was only half of their expression variation across the RILs (Table 4), and ranged from 10 % to 98 % (Additional file 24: Table S14). This difference was due to large differences in the detection ratio and strength of *cis*-eQTLs between global and changed-tissues MZDE genes. Forty-nine out of 57 global MZDE genes (86.0 %) were controlled by strong *cis*-eQTL, while only 54 % (113/211) of changed-tissues MZDE genes were controlled by moderate *cis*-eQTLs (Table 4). In other words, expression difference of a global DE gene was attributable to its own genotype, whereas causation of changed-tissues DE gene expression difference was not simple.

**Table 4:** eQTL of global and changed-tissues MZDE genes

	Global	Changed-tissues
MZDE genes detected in 72 hr seedling arrays	95	273
TIGR loci in MZDE genes	68	250
eQTL detected MZDE genes	57	211
Average of explained variation by detected eQTLs	85.6 ± 16.6 %	51.8 ± 31.4 %
*cis*-eQTL detected MZDE genes	49	113
Average of explained variation by detected *cis*-eQTL	86.5 ± 16.6 %	68.7 ± 28.2 %

Even where *cis*-eQTLs were detected for changed-tissues MZDE genes, the sums of explained variation were variable (Additional file 24: Table S14). We investigated the relationship between sums of explained variation by the detected eQTLs and Minghui63 *τ* for changed-tissues MZDE genes with and without *cis*-eQTLs (Fig. 7b). Changed-tissues MZDE genes were separated into two groups; in the first group expression variations were well explained (>80 %), most of genes had *cis*-eQTL, and the *τ* range was divergent. In the other group, expression variations were not explained (<40 %) by the detected eQTLs, most of genes did not have *cis*-eQTLs, and *τ* was high, > 0.7 (Fig. 7b). We also investigated the relationship between the sums of explained variation and Zhenshan97 *τ* (Additional file 25: Figure S11). The tendency of the two groups separation was the same. For 39 genes, the differences between Minghui63 *τ* and Zhenshan97 *τ* were more than 20 % of the means of the two *τ*, however, the sums of explained variation for these genes were very high and they belonged to the first group (Additional file 24: Table S14). It is noteworthy that many DE genes with high *τ*, where expression evolution is rapid, were not regulated by *cis*-eQTLs and expression variations were not explained by the few detected QTLs. We also checked whether these tissue specifically expressed changed-tissues MZDE occurred at the highest expression tissue or not. We calculated relative expression intensity to the highest intensity that was used for *τ* calculation. Most of the relative expression intensities of eQTL detected changed-tissues MZDE genes did not exceed 95 % in either Minghui63 or Zhenshan97 early seedling stage (Additional file 24: Table S14). Therefore these changed-tissues MZDEs did not occur in their highest expression tissue.

## Discussion

Most studies of expression evolution have not discriminated between contrasting types of DE genes. Using expression atlas data, we could classify *japonica-indica* differentially expressed (*ji*DE) genes into two different types, global *ji*DE genes and changed-tissues *ji*DE genes. They had contrasting characteristics in detected numbers, tissue expression specificity, sequence evolution, distribution in genome, and types of genes as shown in Table 2.

Many microarray studies have focused on relationships between sequence evolution and expression evolution leading to controversial conclusions in different species (reviewed in [7, 8]). In *Drosophila* [25–27], in fire ants [28], between human and chimpanzee [29], between human and mouse [30, 31], and in *Arabidopsis*, rice and maize [32], correlations between gene sequence evolution and gene expression evolution were observed. However, Tirosh and Barkai (2008) observed no correlation in yeast [33], and in contrast to Yang and Wang [32], Movahedi et al. observed no correlation between sequence and expression evolution in *Arabidopsis* and rice [34]. Some possible explanations for this inconsistency relate to our observations that sequence evolution of global type DE genes seemed to be rapid and that of changed-tissues type DE gene was not. There are some difficulties in the evaluation of expression evolution in relation to sequence evolution. One difficulty comes from tissue and cell type dependency of gene expression. If a gene is SE in an observed tissue, it may be DE in another tissue. If a gene’s expression in species A is higher than in species B in one tissue, the gene could be more highly expressed in species B than species A in another tissue. Studies of Drosophila [25–27] and in fire ants [28] used whole body RNA, and it is possible that tissue dependent changed-tissues type DEs were concealed and tissue independent global type DEs were emphasized. In mammals [29–31], since cell type compositions in some tissues were different among distant species, cell type dependent changed-tissues DEs would be concealed, and cell type independent global DEs would be emphasized. In addition to differences in cell type composition, it is difficult to evaluate gene expressions among different platform microarray data of distant species [31, 35]. Probe sensitivities depend on their sequences. Signal intensity of each probe within a probe set targeting the same transcript often varied more than 10-folds, log2-intensity > 3 (for example see Additional file 1: Figure S1). The large gene expression divergences of diverged sequence genes observed in *Arabidopsis*, rice and maize by Yang and Wang [32] might therefore be overestimated. On the other hand, Movahedi et al*.* [34] studied the correlated gene expression of 4630 orthologous gene pairs between *Arabidopsis* and rice, and concluded no significant correlation between gene sequence and gene expression evolution. Since global type DE genes were free from negative selection and may have rapid changes in their sequence, in distant species the orthologs of such genes may be absent precluding observation of their expression changes.

Jung et al. [36] detected 490 *japonica* preferentially expressed genes and 104 *indica* preferentially expressed genes using 983 Affymetrix rice array data sets that comprising 595 *indica* data sets and 388 *japonica* data sets from public databases. Since they ignored tissue type in their analysis, detected *ji*DE genes were all global type *ji*DE genes.

Davidson et al. [37] performed RNA-seq in eight visually similar developmental stages of reproductive tissues and leaves from *Brachypodium distachyon*, *Sorghum bicolor*, and rice, whose last common ancestor was 45–60 mya [38–41]. They observed that there was no significant enrichment of diverged expressions for higher *K*_a_/*K*_s_ orthologs [37]. This observation was the same as in the array study between rice and Arabidopsis [34], and changed-tissues type DE genes in our study.

There are contradictory reports that broadly expressed genes have either more conserved expression [29] or less conserved expression [31] than specifically expressed genes. Our observations were that the number of global DE genes was independent of expression tissue specificity and that changed-tissues DE genes were over-represented among tissue specifically expressed genes (Fig. 2b). Liao and Zhan [31] measured expression divergence between human and mouse genes in 26 tissues calculating Pearson’s correlation coefficient between them as if there was no silent tissue, and overall expressions of tissue specifically expressed genes were more similar than those of broadly expressed genes. As mentioned by Piasecha et al*.* [35], both Euclidean distance and Pearson’s correlation coefficient of expression intensities are dependent on expression tissue specificity, and underestimate expression divergence of specifically expressed genes, because many silent or low expressed tissues cause similar overall expression between tissue specifically expressed homologous genes.

TE-related genes were over-represented in global *ji*DE genes and *cis*-QTL mainly regulated global MZDE genes. More than half of TE-related genes were differentially methylated between *japonica* Nipponbare and *indica* 93*-*11 [42]. Epigenetic differences in *cis*-regulatory regions and within genes would make a large contribution to global DE genes. We did not observe negative selection by pleiotropic effects on global *ji*DE genes, because global *ji*DE genes were not enriched in tissue specifically expressed genes.

For changed-tissues *ji*DE genes, we cannot evaluate negative selection by pleiotropic effects, because we did not evaluate overall expression divergence in all tissues. A simple stochastic model does not explain three phenomena concerning changed-tissues *ji*DE genes with higher *τ*: rapid expression evolution, DE gene enrichment, and unexplained variation by detected eQTLs. The most simple stochastic model to explain rapid expression evolution and DE gene enrichment in higher *τ* is that more factors or sites are involved in the expression regulation of tissue specifically expressed genes than for broadly expressed genes, and the probability of DE in tissue specifically expressed genes is higher than that in broadly expressed genes. Analysis of eQTL does not aim at the detection of potential loci for gene regulation but at detection of causal loci, which are genetically or epigenetically different between the parents, for gene expression variations across the population. There are mainly three reasons for unexplained variation by detected eQTLs. The first one is low heritability of the gene expression. The second one is that the gene expression is regulated by multiple small effect eQTLs that are not detectable with a small population size. The last one is that the gene expression is regulated by strong epistasis among eQTLs. For the first case, since SNEP took variance within replicates into consideration for DE detection [18], the heritability of DE detected by SNEP must be high. Contrary to the simple stochastic model, the second and last cases require multiple loci changes for a single DE gene. This would be suggestive of positive selection for DE in tissue specifically expressed genes.

## Conclusions

We expected different evolutionary forces to act on different types of gene expression alteration, and that different types of genes might show different types of differential expression between species. Conforming to this expectation, there were two contrasting types of DE genes, which we designated global DE genes and changed-tissues DE genes. Expression differences of most global DE genes were regulated by *cis*-eQTLs. The number of global DE genes expressed in each tissue was relatively constant, and occurrence of global DE alteration was independent of gene expression tissue specificity (*τ*). Molecular evolution of some global DE genes was free from constraints. Some global DE genes were clustered on the genome, and TE related genes and disease resistance genes were enriched among global DE genes. Meanwhile, changed-tissues DE genes were separated into two groups considering their regulation of differential expression and *τ*. In the first group expression variations were well explained by the detected both *cis*-eQTLs and *trans*-eQTLs. In the other group, most of the genes did not have *cis*-eQTLs and regulations of the differential expression of these genes was complicated, and *τ* was high. Expression evolution of changed-tissues DE genes with high *τ* was rapid. Changed-tissues DE genes were overrepresented among higher *τ* genes, the number of changed-tissues DE genes increasing linearly with the abundance of tissue specifically expressed genes. The constraints on molecular evolution of changed-tissues DE genes were the same as those for SE genes.

The two contrasting types of DE genes provide possible explanations for the previous controversial conclusions about the relationships between molecular evolution and expression evolution of genes in different species, and the relationship between expression breadth and expression conservation in evolution.

## Methods

### Data for this study

All Affymetrix rice expression data were downloaded from the Gene Expression Omnibus (GEO) at http://www.ncbi.nlm.nih.gov/geo/ (Additional file 2: Table S1). We obtained 121 and 200 Affymetrix CEL files for *japonica* and *indica*, respectively [16–19]. No ethics approval was required for the public microarray data used in this study.

### Reconstruction of array probe sets that uniquely hybridize to single transcripts

The Affymetrix Rice Genome array was designed before the completion of rice genome sequencing efforts [14] and several probes on the array can potentially hybridize to non-target genes or are targeted to introns, according to updated annotations. To eliminate these possibilities, we reconstructed array probe sets using three annotations, TIGR Rice annotation version 5.0 (TIGR5) [21], the Rice Annotation Project (RAP2) [22], and the target transcripts used to design the rice genome array (Affy). The Affymetrix Rice Genome array has 57,381 probe sets (including 187 control probe sets) containing ~631,366 PM probes/features. Removing probes designed for pre-labeled hybridization spiked-in controls, a total of 629,092 probes were extracted for rice expression analysis and their sequences were aligned to TIGR5 and RAP2 using by BLASTN analysis [43]. Each PM probe is 25 bp long and the complete match score to target sequence is 25, and gap open, gap extend and mismatch penalty are 5, 2 and 3, respectively as previously used in Horiuchi et al*.* [19]. We removed probes, which hit multiply against target sequences with a score greater than 17. To distinguish whether genes of TIGR5, RAP2, and Affy were the same locus on Nipponbare genome or not, 66,710 transcripts of TIGR5 “all.cDNA”, 53,461 transcripts of RAP2 “all_nuc” and ‘prediction_nuc’, and also 52,350 Affy target transcripts were aligned on the IRGSP v.4 Nipponbare sequence (http://rgp.dna.affrc.go.jp/IRGSP/) by BLAT search [44]. If a transcript were aligned multiple locations with more than 80 % matches, or a probe hit multiple transcripts from different annotation projects mapped on different locations, these hit probes were eliminated. When we selected a gene model from different annotations at the same location on the IRGSP v4 genome, we made the priority order of annotations as follows; TIGR5, RAP2, and Affy. 45,408 unique target transcripts targeted by the remained 521,437 unique probes were selected (Additional file 26: Table S15). Subsequently the 45,408 unique target transcripts were also aligned on the *indica* cultivar 93-11 genome (BGI version 2003-08-01 http://rice.genomics.org.cn/rice/index2.jsp) by BLAT search [44], and to investigate whether the 521,437 probes were unique on 93-11 genome, the 521,437 unique probes were aligned on the 93-11 transcript sequences that were extracted from BLAT search with more than 80 % matches. After elimination of 146 transcripts with multiple hits on the 93-11 genome, 510,458 unique probes of 45,035 transcripts survived uniquely targeting both the Nipponbare and 93-11 genomes. For robust evaluation of transcript expression level, we eliminated 1101 transcripts that were targeted by less than three unique probes, and we used 508,908 unique probes of 43,934 transcripts in this study (Additional file 26: Table S15).

### Calculation of expression value and detection of DE genes

All statistical analyses were performed using Bioconductor packages (http://www.bioconductor.org/) in R (http://www.r-project.org/). The ‘.CEL’ files were read directly into R using “affy” package and Perfect match (PM) probe intensities of the 508,908 unique probes for the unique transcripts were extracted and transformed to log_2_-value. Quantile normalization of the 508,908 probes in the 321 arrays was performed using “normalize.quantiles” in “preprocessCore” package, and the normalized values were used in the following analyses.

We used a modified SNEP two-side test for unequal replications [18] (http://www.ism.ac.jp/~fujisawa/SNEPunequal/index.html) to calculate both *p* values of probes for testing null hypothesis of SFPs and *t* values of transcript for testing null hypothesis of expression polymorphism, simultaneously.

### Hierarchical cluster analysis of 321 arrays

The expression values of the 12,097 genes that were *ji*SE between Nipponbare and 93-11 in shoot and panicle were estimated as median intensities after elimination of SFP probes detected between Nipponbare and 93-11, and *Z* scaled across the 321 arrays by using the “genefilter” package. For clustering analysis, the centered Pearson’s correlation distances for each gene were calculated by using the “amap” package, and the dissimilarity structure was produced by the agglomeration method “ward” linkage clustering in “stats” package. To visualize hierarchical cluster analysis, the “gplots” package was used.

### Determination of silent genes and calculation of Yanai’s tissue specificity index

In determination of silent genes and calculation of Yanai’s tissue specificity index *τ* [23] for each strain, we eliminated 63,502 SFP probes that were detected between the three combinations, NM, NZ, and MZ, in the five tissues by SNEP analysis, and could use 43,932 genes expression values, which were evaluated from median intensities in the respective probe set. On determination of silent genes in each strain, we used 44 Nipponbare tissues, 36 Minghui63 tissues and 39 Zhenshan97 tissues. If log_2_-intensities of a gene did not exceed 7 in all 44 Nipponbare tissues but were highly expressed in any of 36 Minghui63 or 39 Zhenshan97 tissues, we called the gene as a Nipponbare silent gene. Minghui63 silent genes and Zhenshan97 silent genes were also defined in the same way. We calculated Nipponbare *τ*, Minghui63 *τ*, and Zhenshan97 *τ* By the following equation using the respective tissue data sets;

$$ \tau =1-\frac{{\displaystyle {\sum}_{i=1}^N}\left(1-{x}_i\right)}{N-1} $$

Where *N* is the number of tissues and *x*_*i*_ is a normalized expression value for each tissue divided by the maximum expression intensity in all tissues.

### Determination of *indica* ortholog and calculation of *K*_a_/*K*_s_

We searched *indica* orthologs of all 43,932 Nipponbare transcripts by BLAT search on the 93-11 genome (BGI version 2003-08-01 http://rice.genomics.org.cn/rice/index2.jsp). If a transcript was aligned on the 93-11 genome with more than 80 % match of the transcript, we consider it as the *indica* ortholog. Nipponbare open reading frames (ORF) (“all.cds” from TIGR5, “all_orf_nuc” and “rep_orf_nuc” from RAP2) were investigated for “n” content, genes having ‘n’ were removed, and 35,293 transcripts had complete Nipponbare ORFs. Of these, 32,465 genes had a putative *indica* ortholog according to BLAT search. The Nipponbare ORFs and their corresponding 93-11 transcripts were aligned by BLASTN to predict 93-11 ORFs, and the predicted 93-11 ORFs were conceptually translated to their amino acid sequences. Pair-wise alignment between Nipponbare and 93-11 protein sequences was performed using BLASTP. 22,324 protein lengths were same between Nipponbare and 93-11. *Ka*/*Ks* values were calculated as molecular evolution rates for putative ortholog pairs of the same protein length by using “seqinr” package in R.

### Gene ontology enrichment analyses

For performing GO enrichment analyses, gene ontologies of Gramene Release 34-October 2011, in which 30,634 *japonica* transcripts were assigned to 160,347 GO terms were downloaded via GrameneMart of GRAMENE (http://www.gramene.org/). A total of 3711 GO terms were assigned for 19,053 out of 43,932 genes we used, and 1394 GO terms had more than 5 genes. For these 1394 GO terms, we investigated whether particular GO terms were enriched in an objective gene list using Fisher’s exact test.

### Availability of supporting data

The reconstructed probe set consisting of the AffyID, ProbeNumber, NewTranscriptName, and NewProbeNumber for the 508,908 unique probes is available online at http://www.shigen.nig.ac.jp/rice/oryzabase/asset/supplementalData/harushima/supplemental_data_S1.txt. An R script for performing a modified SNEP two-side test for unequal replications is available at http://www.ism.ac.jp/~fujisawa/SNEPunequal/index.html.

## Additional files

Additional file 1: Figure S1. Probe Selection by using SNEP two-sided test. (PDF 313 kb) (PDF 301 kb) Additional file 2: Table S1. Rice gene expression data sets. (XLSX 70 kb) (XLSX 66 kb) Additional file 3: Figure S2. Dendrogram of 321 arrays from *japonica* Nipponbare, three *indica* varieties 93-11, Zhenshan97, and Minghui63. (PDF 340 kb) (PDF 331 kb) Additional file 4: Table S2. Number of highly expressed genes and SNEP detected *ji*DE genes in each tissue in the two *japonica* and *indica* combinations. (DOCX 106 kb) (DOCX 102 kb) Additional file 5: Table S3. Highly expressed genes and the two types of *ji*DE genes of the two *japonica*-*indica* combinations in the five tissues. (XLSX 53 kb) (XLSX 51 kb) Additional file 6: Figure S3. Scatter plots of *τ* values for highly expressed genes in respective *japonica* or *indica* tissues. (PDF 291 kb) (PDF 280 kb) Additional file 7: Figure S4. Overlap of *ji*DE genes detected in the five tissues of NZ combination. (PDF 537 kb) (PDF 521 kb) Additional file 8: Table S4. Molecular evolution of *ji*DE genes. (XLSX 61 kb) (XLSX 62 kb) Additional file 9: Figure S5. Ratio distribution of Nipponbare or Minghui63 highly expressed genes to every 100 mapped genes. (PDF 680 kb) (PDF 662 kb) Additional file 10: Figure S6. Ratio distribution of Nipponbare or Zhenshan97 highly expressed genes to every 100 mapped genes. (PDF 684 kb) (PDF 665 kb) Additional file 11: Figure S7. Ratio distribution of changed-tissues *ji*DE genes of the NM combination. (PDF 762 kb) (PDF 741 kb) Additional file 12: Figure S8. Ratio distribution of changed-tissues *ji*DE genes of the NZ combination. (PDF 754 kb) (PDF 733 kb) Additional file 13: Table S5. Expression of transposable element (TE) related genes. (XLSX 45 kb) (XLSX 44 kb) Additional file 14: Table S6. GO enrichment analysis of changed-tissues and global *ji*DE genes of the NM combination. (XLSX 16 kb) (XLSX 12 kb) Additional file 15: Table S7. GO enrichment analysis of global *ji*DE genes of the NM combination. (XLSX 57 kb) (XLSX 52 kb) Additional file 16: Table S8. GO enrichment analysis of global *ji*DE genes of the NZ combination. (XLSX 53 kb) (XLSX 49 kb) Additional file 17: Table S9. GO enrichment analysis of tissue specific changed-tissues *ji*DE genes of the NM combination. (XLSX 49 kb) (XLSX 47 kb) Additional file 18: Table S10. GO enrichment analysis of tissue specific changed-tissues *ji*DE genes of the NZ combination. (XLSX 45 kb) (XLSX 41 kb) Additional file 19: Figure S9. Expression types of global DE genes among the three strains. (PDF 344 kb) (PDF 333 kb) Additional file 20: Table S11. Expression types of global DE genes among the three strains. (XLSX 49 kb) (XLSX 45 kb) Additional file 21: Figure S10. Commonly detected changed-tissues *ji*DE genes between the NM and NZ combinations. (PDF 385 kb) (PDF 372 kb) Additional file 22: Table S12. Commonly detected changed-tissues *ji*DE genes between the NM and NZ combinations. (XLSX 53 kb) (XLSX 49 kb) Additional file 23: Table S13. Details of expression quantitative trait loci (eQTLs) detected global Minghui63-Zhenshan97 differentially expressed (MZDE) genes. (XLSX 45 kb) (XLSX 17 kb) Additional file 24: Table S14. Details of expression quantitative trait loci (eQTLs) detected changed-tissues Minghui63-Zhenshan97 differentially expressed (MZDE) genes. (XLSX 127 kb) (XLSX 121 kb) Additional file 25: Figure S11. Relationship between explained variation by detected eQTLs and Zhenshan97 τ. (PDF 455 kb) (PDF 442 kb) Additional file 26: Table S15. Description of the number of unique transcripts and unique probes. (DOCX 111 kb) (DOCX 105 kb)

## Abbreviations

Affy: The target transcripts used to design the Affymetrix rice genome array
BAC: Bacterial artificial chromosome
BAC-by-BAC sequencing: A clone-by-clone shotgun sequencing
BLAST: Basic local alignment search tool
BLAT: The BLAST-like alignment tool
DE genes: Differentially expressed genes
eQTL: Expression quantitative trait loci
GEO: Gene expression omnibus
GO: Gene ontology
*ji*DE genes: *Japonica*-*indica* differentially expressed genes
*ji*SE genes: *Japonica*-*indica* similarly expressed genes
*K*_a_: The number of non-synonymous substitutions per non-synonymous site
*K*_s_: The number of synonymous substitutions per synonymous site
MZ combination: Minghui63 and Zhenshan97 combination
MZDE genes: Minghui63-Zhenshan97 differentially expressed genes
NM combination: Nipponbare-Minghui63 combination
NZ combination: Nipponbare-Zhenshan97 combination
ORF: Open reading frame
RAP2: Annotation dataset of the second rice annotation project meeting
RNA-seq: RNA sequencing
SFP: Single feature polymorphisms
SNEP: Simultaneous detection of nucleotide and expression polymorphisms
τ: The tissue specificity index
TE-related genes: Transposable element-related genes
TIGR5: The institute for genome research rice annotation release 5.0.
