# Supplementary material for: Global expression differences and tissue specific expression differences in rice evolution result in two contrasting types of differentially expressed genes
Source: BMC Genomics. 2015 Dec 23;16:1099. doi: 10.1186/s12864-015-2319-1 (PMC4690246; doi:10.1186/s12864-015-2319-1)
Supplement: Additional file 1: Figure S1. — Probe Selection by using SNEP two-sided test. (PDF 313 kb) (PDF 301 kb) [file 12864_2015_2319_MOESM1_ESM.pdf]

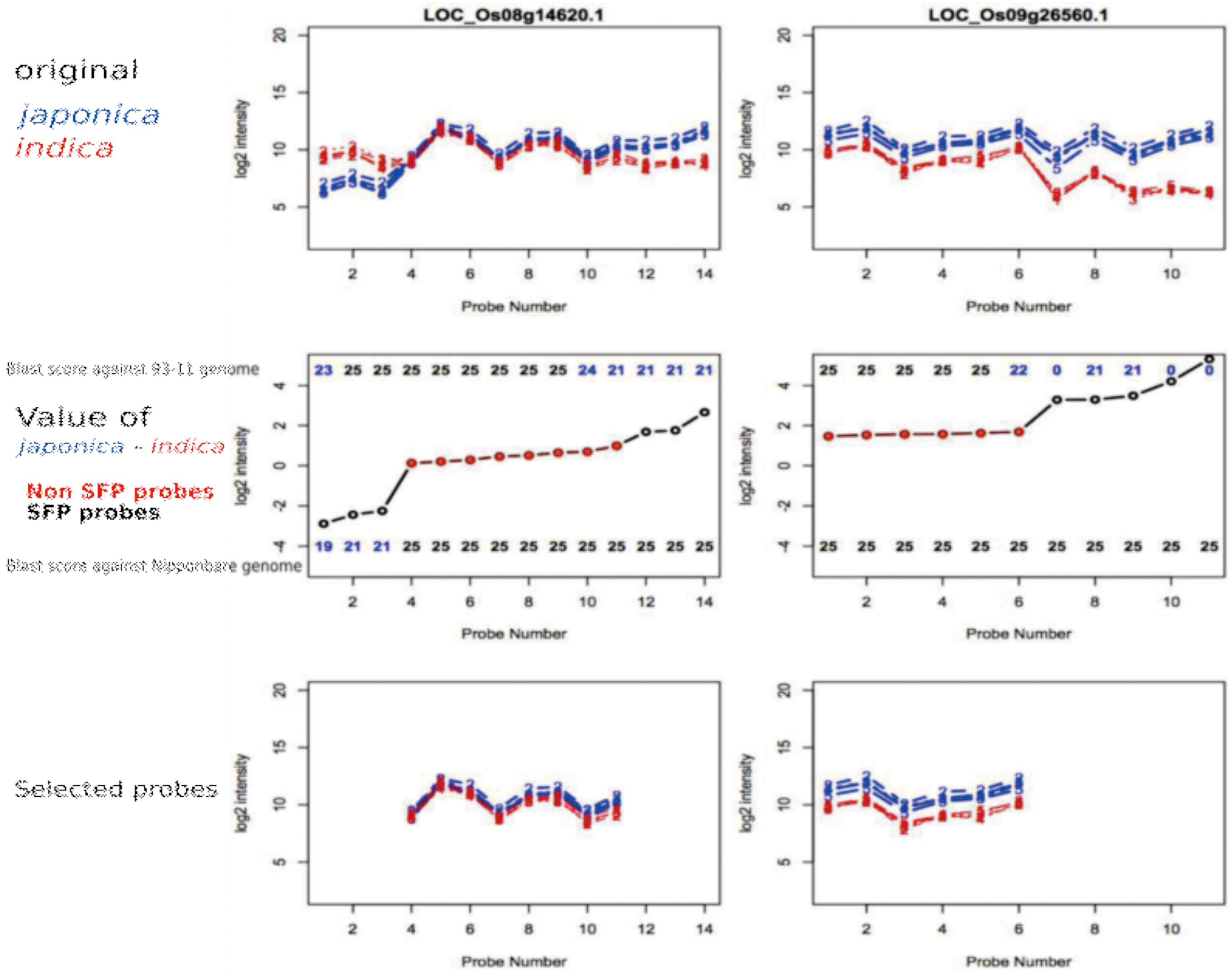

Figure S1. Probe Selection by using SNEP two-sided test. Each transcript expression data was subjected to SNEP two-side analysis. Raw perfect match probe intensities were used. Probes were ordered by differences between *japonica* and *indica*. Values of top, middle, and bottom panels indicated original, difference between *japonica* and *indica*, selected probes to evaluate expression difference by SNEP, respectively. In top and bottom panels, blue and red shows *japonica* and *indica* probe intensities, respectively. In the middle panel, values of non-SFP probes were shown in red, and probes with nucleotide polymorphisms were shown with blue blast scores. In bottom panel, non-SFP probes were only plotted.
