# Supplementary material for: Global expression differences and tissue specific expression differences in rice evolution result in two contrasting types of differentially expressed genes
Source: BMC Genomics. 2015 Dec 23;16:1099. doi: 10.1186/s12864-015-2319-1 (PMC4690246; doi:10.1186/s12864-015-2319-1)
Supplement: Additional file 4: Table S2. — Number of highly expressed genes and SNEP detected jiDE genes in each tissue in the two japonica and indica combinations. (DOCX 106 kb) (DOCX 102 kb) [file 12864_2015_2319_MOESM4_ESM.docx]

**Table S2. Number of highly expressed genes and SNEP detected *ji*DE genes in each tissue in the two *japonica* and *indica* combinations.**

|  | Nipponbare | | Minghui63 | | Nipponbare or Minghui63 | |
| --- | --- | --- | --- | --- | --- | --- |
| Tissue | Expressed^1^ | *ji*DE^2^ | Expressed^1^ | *ji*DE^2^ | Expressed^1^ | *ji*DE^2^ |
| Endosperm | 12,145 | 750 | 12,545 | 648 | 13,893 | 933 |
| Anther | 12,136 | 930 | 12,021 | 796 | 13,901 | 1,102 |
| Panicle | 13,072 | 716 | 13,410 | 562 | 14,600 | 937 |
| Root | 12,671 | 1,017 | 13,132 | 882 | 14,474 | 1,252 |
| Leaf | 12,723 | 1,212 | 13,213 | 996 | 14,486 | 1,476 |
| The five tissues^3^ | 19,710 | 3,500 | 19,996 | 3,173 | 21,810 | 4,226 |

|  | Nipponbare | | Zhenshan97 | | Nipponbare or Zhenshan97 | |
| --- | --- | --- | --- | --- | --- | --- |
| Tissue | Expressed^1^ | *ji*DE^2^ | Expressed^1^ | *ji*DE^2^ | Expressed^1^ | *ji*DE^2^ |
| Endosperm | 12,145 | 671 | 12,480 | 604 | 13,810 | 857 |
| Anther | 12,136 | 596 | 12,648 | 497 | 14,009 | 775 |
| Panicle | 13,072 | 711 | 13,222 | 626 | 14,548 | 973 |
| Root | 12,671 | 888 | 13,159 | 764 | 14,406 | 1,118 |
| Leaf | 12,723 | 1,294 | 12,530 | 931 | 14,142 | 1,494 |
| The five tissues^3^ | 19,710 | 3,097 | 19,420 | 2,765 | 21,431 | 3,794 |

^1^ A gene expression value was defined as the average of replicate median of non-SFP probes log_2_-intensities in a set. We defined highly expressed genes as those having an expression value higher than 7.

^2^ Detected number of *ji*DE genes among the highly expressed genes by SNEP analysis.

^3^ Total number of genes expressed in any of the five tissues.
