# Supplementary material for: Global expression differences and tissue specific expression differences in rice evolution result in two contrasting types of differentially expressed genes
Source: BMC Genomics. 2015 Dec 23;16:1099. doi: 10.1186/s12864-015-2319-1 (PMC4690246; doi:10.1186/s12864-015-2319-1)
Supplement: Additional file 6: Figure S3. — Scatter plots of τ values for highly expressed genes in respective japonica or indica tissues. (PDF 291 kb) (PDF 280 kb) [file 12864_2015_2319_MOESM6_ESM.pdf]

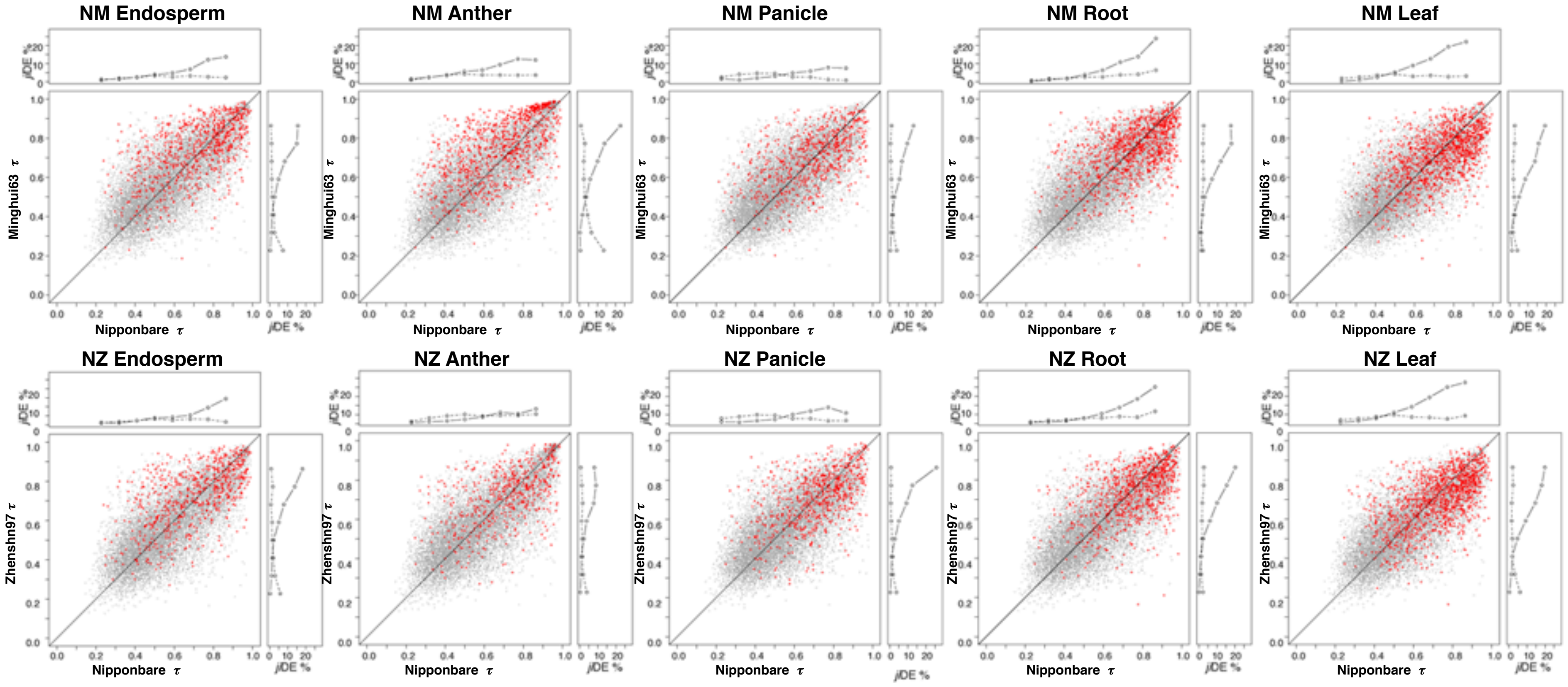

Figure S3. Scatter plots of τ values for highly expressed genes in respective *japonica* or *indica* tissues. Nipponbare τ , Minghui63 τ , and Zhenshan97 τ values were calculated using 44, 36, and 39 tissues, respectively. Red points represent changed-tissues *ji*DE genes. Diagonal lines means same *japonica* and *indica* τ values. The top and right panels of each scatter plot show the percentages of changed-tissues (solid line) and global (broken line) *ji*DE genes on a window of 0.1 τ window for highly expressed genes in Nipponbare and *indica* strain tissues, respectively.
