# Supplementary material for: Global expression differences and tissue specific expression differences in rice evolution result in two contrasting types of differentially expressed genes
Source: BMC Genomics. 2015 Dec 23;16:1099. doi: 10.1186/s12864-015-2319-1 (PMC4690246; doi:10.1186/s12864-015-2319-1)
Supplement: Additional file 7: Figure S4. — Overlap of jiDE genes detected in the five tissues of NZ combination. (PDF 537 kb) (PDF 521 kb) [file 12864_2015_2319_MOESM7_ESM.pdf]

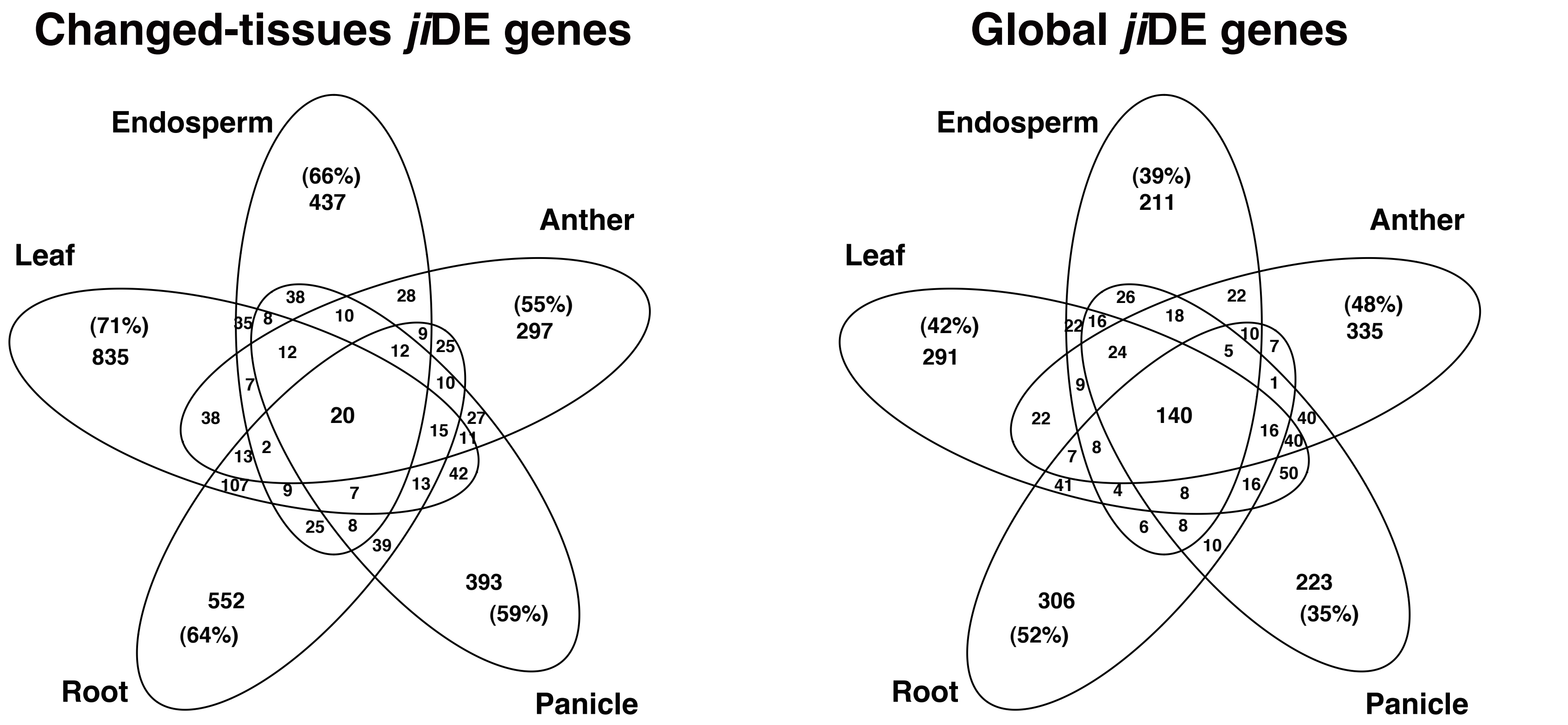

Figure S4. **Overlap of *ji*DE genes detected in the five tissues of NZ combination.** Percentages of tissue specifically detected *ji*DE genes in each tissue were indicated in parentheses.
