# Supplementary material for: Global expression differences and tissue specific expression differences in rice evolution result in two contrasting types of differentially expressed genes
Source: BMC Genomics. 2015 Dec 23;16:1099. doi: 10.1186/s12864-015-2319-1 (PMC4690246; doi:10.1186/s12864-015-2319-1)
Supplement: Additional file 9: Figure S5. — Ratio distribution of Nipponbare or Minghui63 highly expressed genes to every 100 mapped genes. (PDF 680 kb) (PDF 662 kb) [file 12864_2015_2319_MOESM9_ESM.pdf]

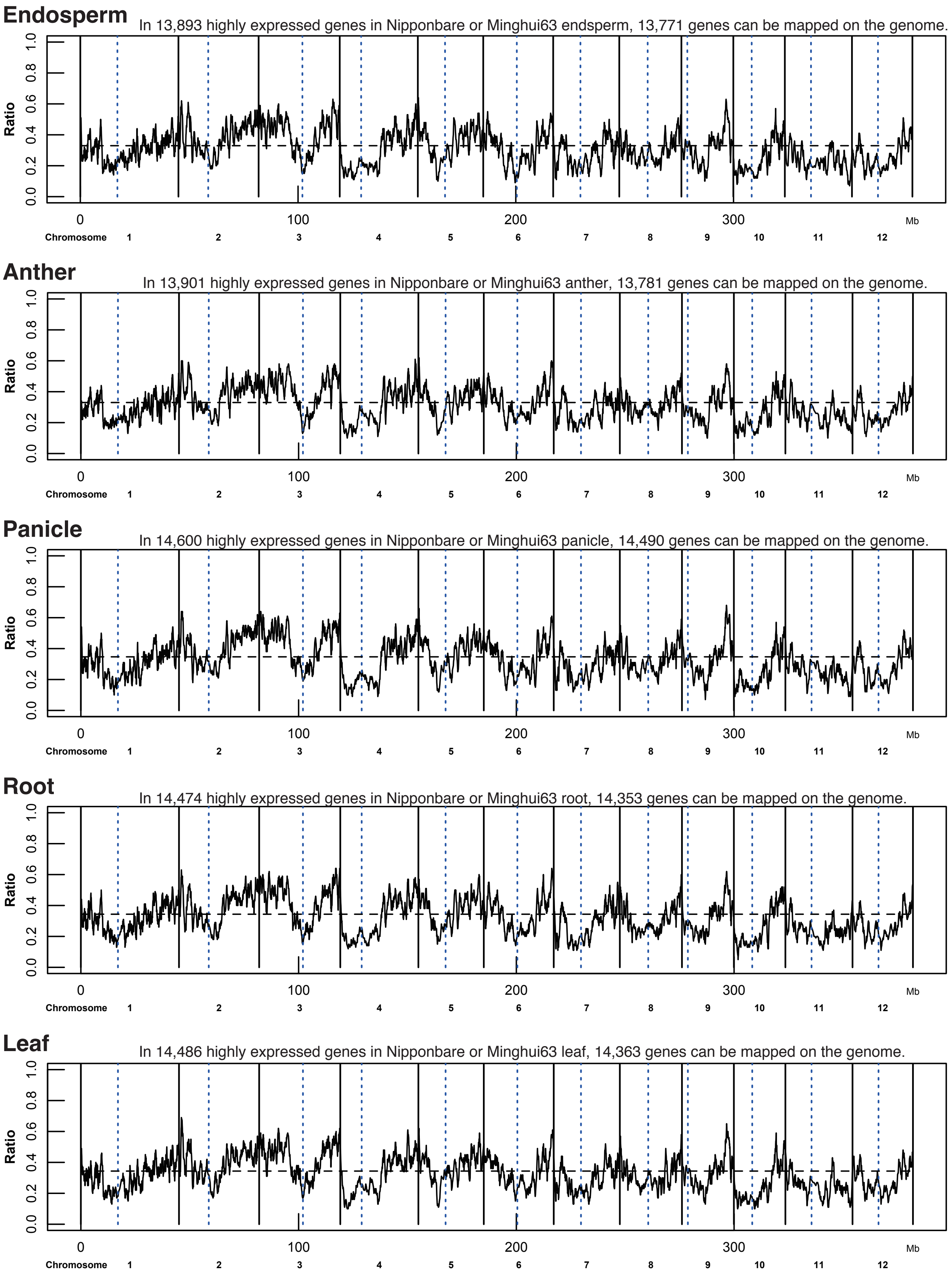

Figure S5. Ratio distribution of Nipponbare or Minghui63 highly expressed genes to every 100 mapped genes.
