# Supplementary material for: Global expression differences and tissue specific expression differences in rice evolution result in two contrasting types of differentially expressed genes
Source: BMC Genomics. 2015 Dec 23;16:1099. doi: 10.1186/s12864-015-2319-1 (PMC4690246; doi:10.1186/s12864-015-2319-1)
Supplement: Additional file 10: Figure S6. — Ratio distribution of Nipponbare or Zhenshan97 highly expressed genes to every 100 mapped genes. (PDF 684 kb) (PDF 665 kb) [file 12864_2015_2319_MOESM10_ESM.pdf]

Endosperm In 13,810 highly expressed genes in Nipponbare or Zhenshan97 endsperm, 13,681 genes can be mapped on the genome.

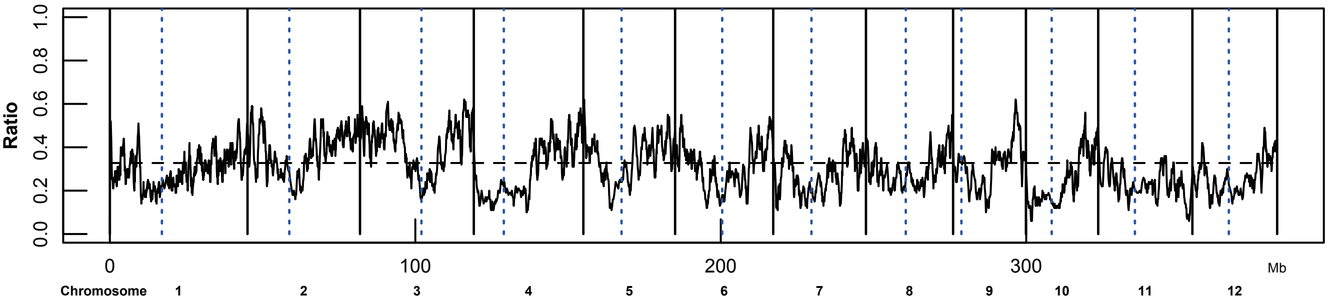

Anther In 14,009 highly expressed genes in Nipponbare or Zhenshan97 anther, 13,885 genes can be mapped on the genome.

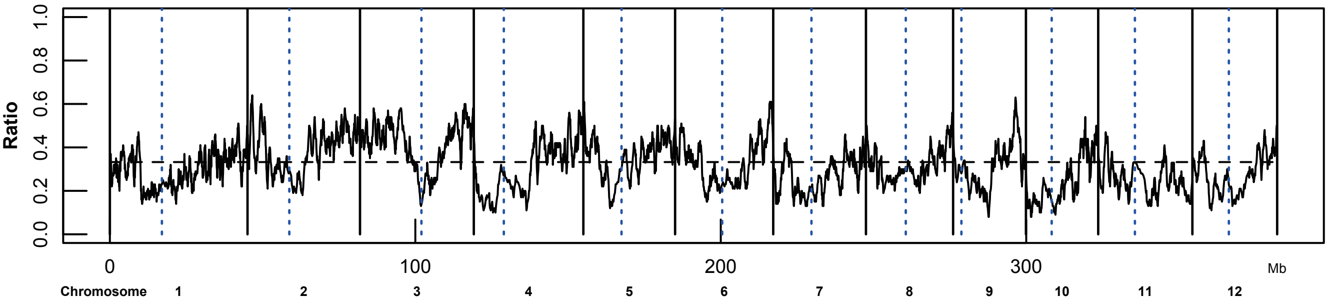

Panicle In 14,548 highly expressed genes in Nipponbare or Zhenshan97 panicle, 14,435 genes can be mapped on the genome.

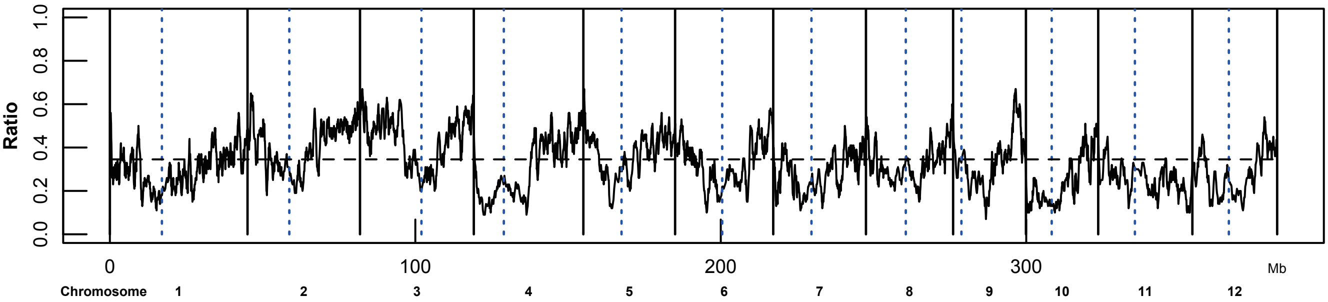

Root In 14,406 highly expressed genes in Nipponbare or Zhenshan97 root, 14,281 genes can be mapped on the genome.

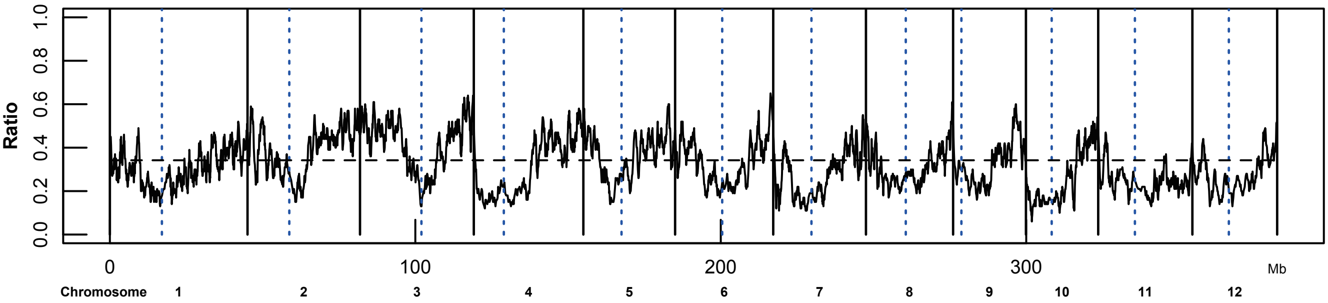

Leaf In 14,142 highly expressed genes in Nipponbare or Zhenshan97 leaf, 14,019 genes can be mapped on the genome.

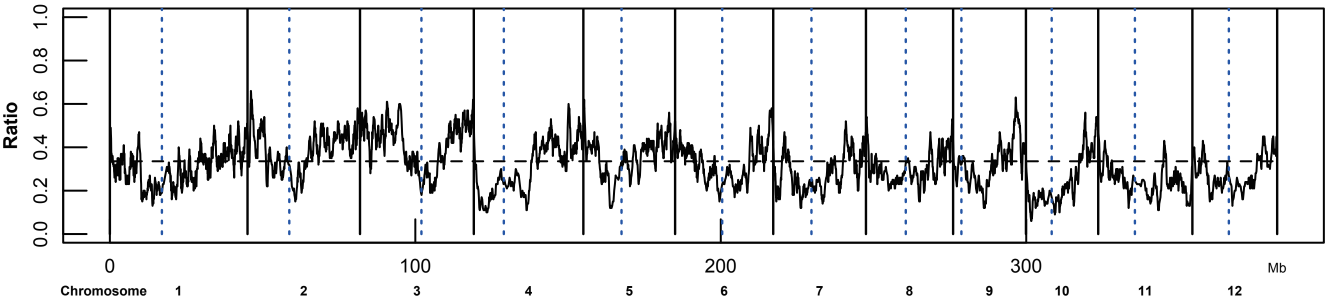

Figure S6. Ratio distribution of Nipponbare or Zhenshan97 highly expressed genes to every 100 mapped genes.
