# Supplementary material for: Global expression differences and tissue specific expression differences in rice evolution result in two contrasting types of differentially expressed genes
Source: BMC Genomics. 2015 Dec 23;16:1099. doi: 10.1186/s12864-015-2319-1 (PMC4690246; doi:10.1186/s12864-015-2319-1)
Supplement: Additional file 11: Figure S7. — Ratio distribution of changed-tissues jiDE genes of the NM combination. (PDF 762 kb) (PDF 741 kb) [file 12864_2015_2319_MOESM11_ESM.pdf]

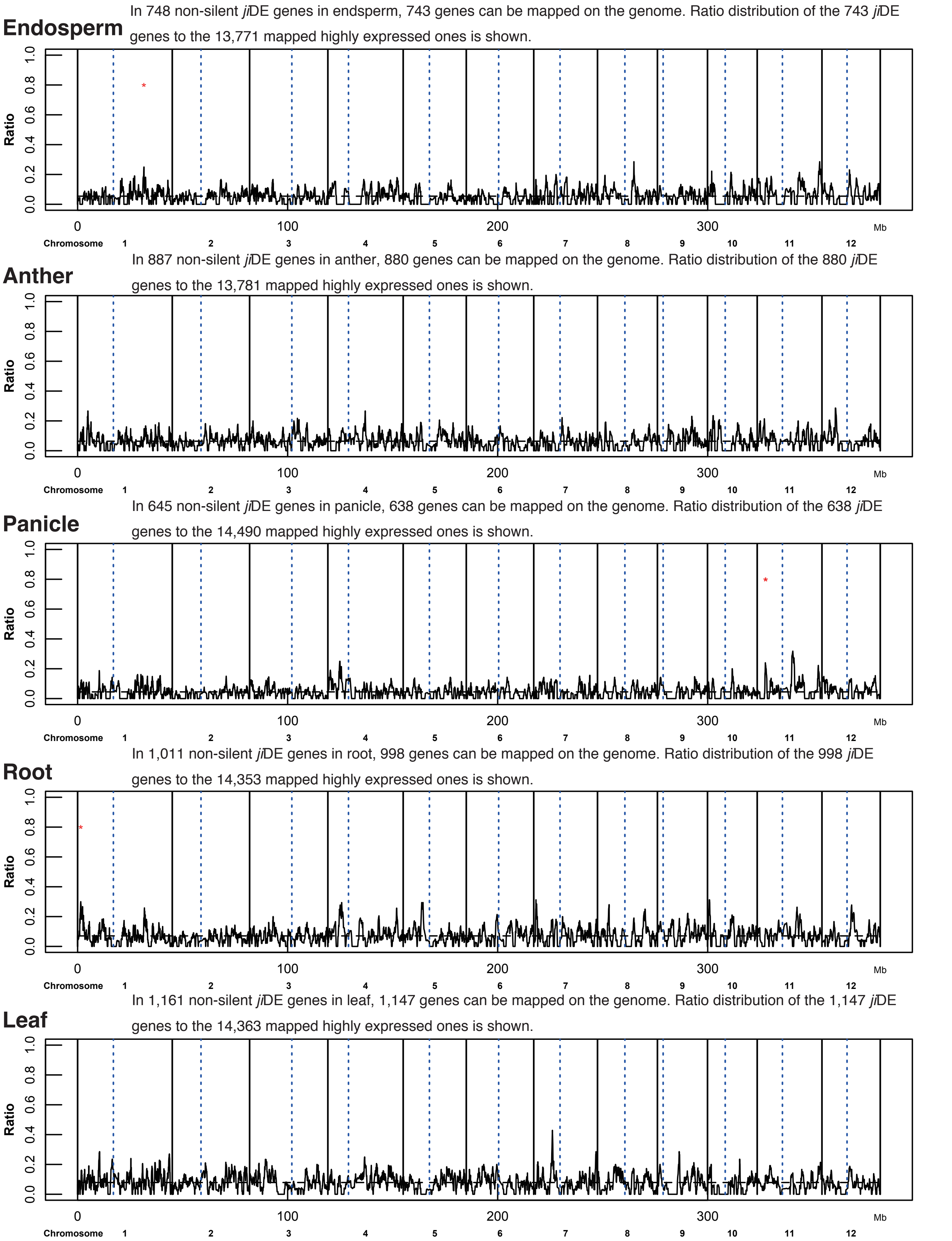

Figure S7. Ratio distribution of changed-tissues *ji*DE genes of the NM combination. Ratios of changed-tissues *ji*DE genes to highly expressed genes in Nipponbare or Minghui63 tissues were plotted for every 100 mapped genes. Red asterisks (*) mean an exceptional ratio that was more than 3 times higher than the 3rd quartile value of the changed-tissues *ji*DE gene ratio distribution, where highly expressed genes were more than the first quartile value of the highly expressed gene rate distribution in 100 mapped genes.
