# Supplementary material for: Global expression differences and tissue specific expression differences in rice evolution result in two contrasting types of differentially expressed genes
Source: BMC Genomics. 2015 Dec 23;16:1099. doi: 10.1186/s12864-015-2319-1 (PMC4690246; doi:10.1186/s12864-015-2319-1)
Supplement: Additional file 12: Figure S8. — Ratio distribution of changed-tissues jiDE genes of the NZ combination. (PDF 754 kb) (PDF 733 kb) [file 12864_2015_2319_MOESM12_ESM.pdf]

Endosperm

In 667 non-silent *ji*DE genes in endsperm, 661 genes can be mapped on the genome. Ratio distribution of the 661 *ji*DE genes to the 13,681 mapped highly expressed ones is shown.

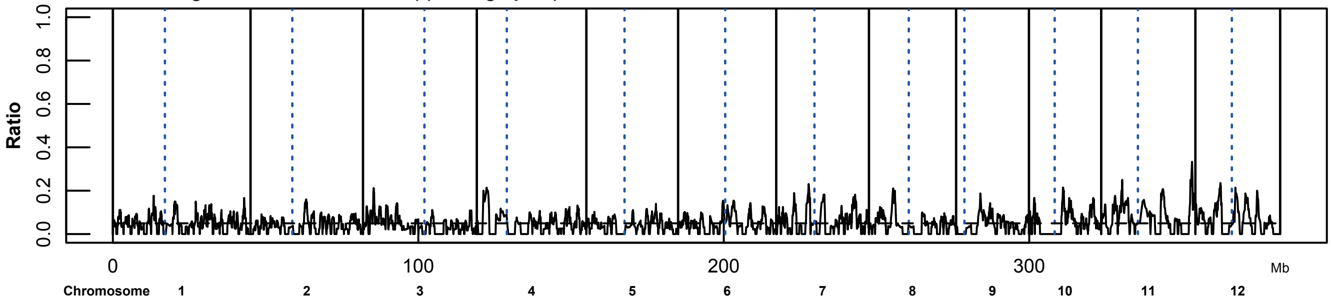

Anther

In 536 non-silent *ji*DE genes in anther, 527 genes can be mapped on the genome. Ratio distribution of the 527 *ji*DE genes to the 13,885 mapped highly expressed ones is shown.

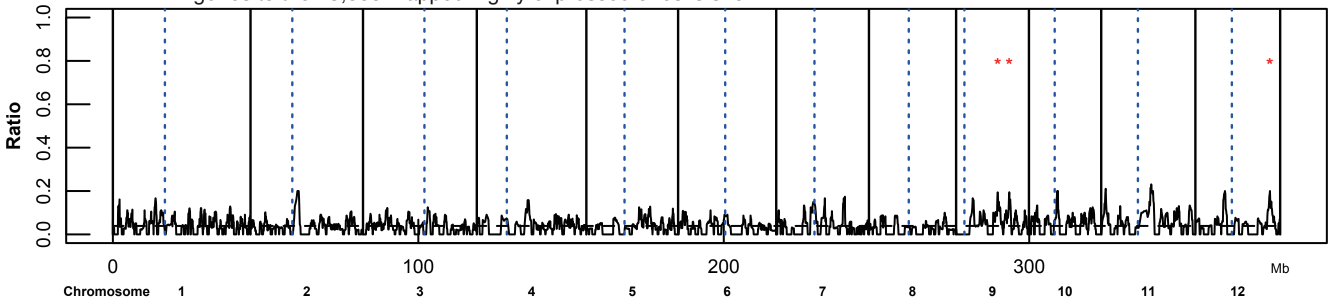

Panicle

In 665 non-silent *ji*DE genes in panicle, 658 genes can be mapped on the genome. Ratio distribution of the 658 *ji*DE genes to the 14,435 mapped highly expressed ones is shown.

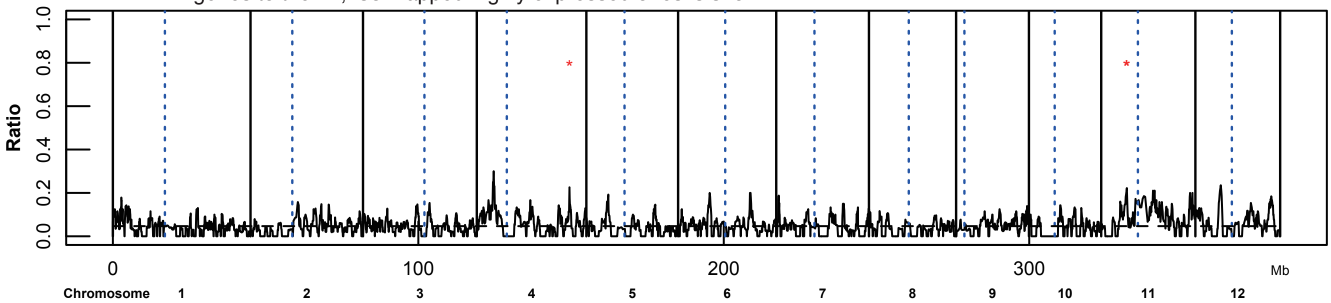

Root

In 866 non-silent *ji*DE genes in root, 855 genes can be mapped on the genome. Ratio distribution of the 855 *ji*DE genes to the 14,281 mapped highly expressed ones is shown.

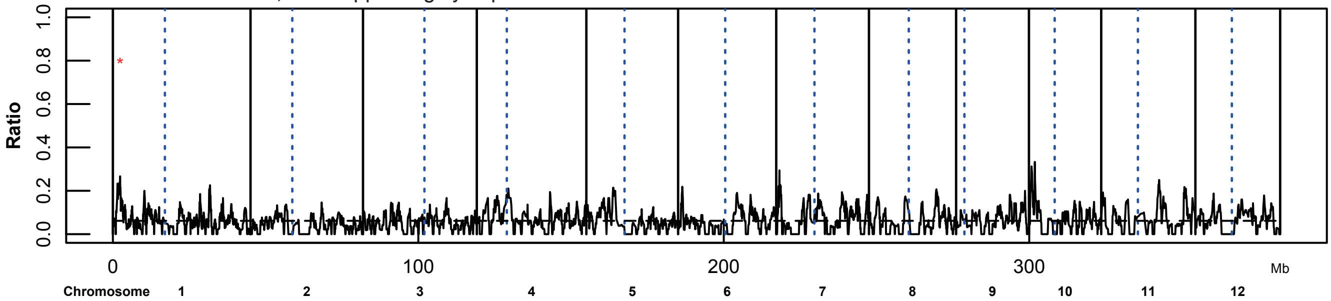

Leaf

In 1,174 non-silent *ji*DE genes in leaf, 1,161 genes can be mapped on the genome. Ratio distribution of the 1,161 *ji*DE genes to the 14,019 mapped highly expressed ones is shown.

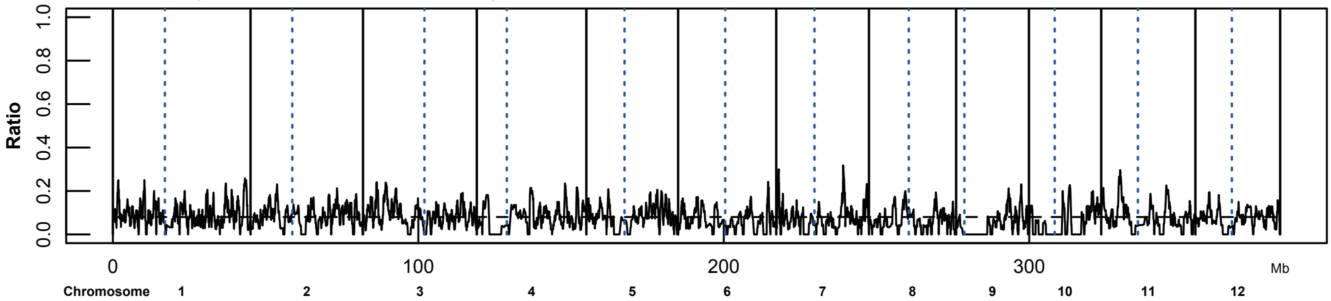

Figure S8. Ratio distribution of changed-tissues *ji*DE genes of the NZ combination. Ratios of changed-tissues *ji*DE genes to highly expressed genes in Nipponbare or Zhenshan97 tissues were plotted for every 100 mapped genes. Red asterisks (*) mean an exceptional ratio that was more than 3 times higher than the 3rd quartile value of the changed-tissues jiDE gene ratio distribution, where highly expressed genes were more than the first quartile value of the highly expressed gene rate distribution in 100 mapped genes.
