# Supplementary material for: Global expression differences and tissue specific expression differences in rice evolution result in two contrasting types of differentially expressed genes
Source: BMC Genomics. 2015 Dec 23;16:1099. doi: 10.1186/s12864-015-2319-1 (PMC4690246; doi:10.1186/s12864-015-2319-1)
Supplement: Additional file 19: Figure S9. — Expression types of global DE genes among the three strains. (PDF 344 kb) (PDF 333 kb) [file 12864_2015_2319_MOESM19_ESM.pdf]

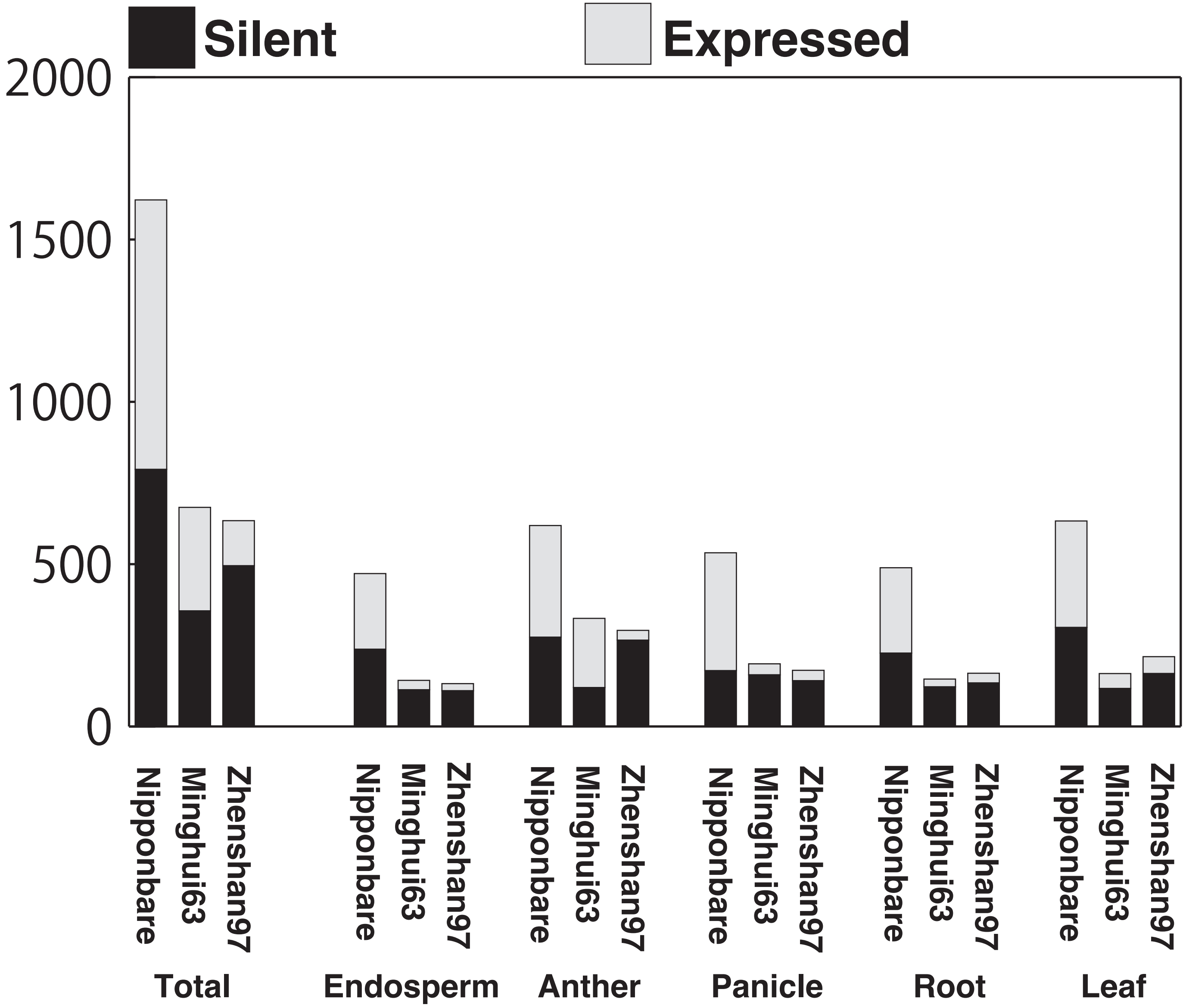

Figure S9. Expression types of global DE genes among the three strains. "Silent" means genes were silent in only one strain but not silenced in the other strains and highly expressed in either of the other stains. "Expressed" means genes were expressed in only one strain but silent in the other strains.
