# Supplementary material for: Global expression differences and tissue specific expression differences in rice evolution result in two contrasting types of differentially expressed genes
Source: BMC Genomics. 2015 Dec 23;16:1099. doi: 10.1186/s12864-015-2319-1 (PMC4690246; doi:10.1186/s12864-015-2319-1)
Supplement: Additional file 21: Figure S10. — Commonly detected changed-tissues jiDE genes between the NM and NZ combinations. (PDF 385 kb) (PDF 372 kb) [file 12864_2015_2319_MOESM21_ESM.pdf]

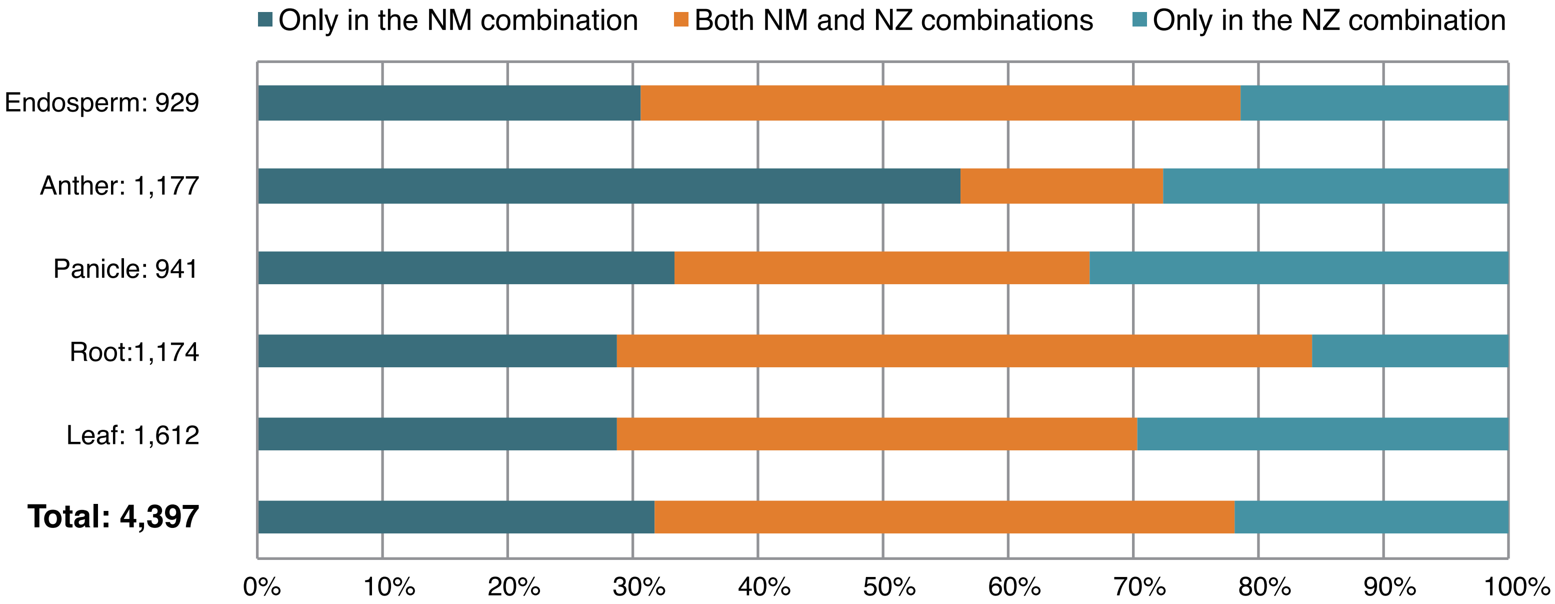

Figure S10. Commonly detected changed-tissues *ji*DE genes. Proportions of three kinds of changed-tissues *ji*DE genes were shown. **Dark blue bars** indicate *ji*DE was observed only in the NM combination. **Dark beige bars** indicate *ji*DE was observed in both NM and NZ combinations. **Light blue bars** indicate *ji*DE was observed only in the NZ combination. Numbers in the tissue labels are total numbers of changed-tissues *ji*DE genes.
