# Supplementary material for: Global expression differences and tissue specific expression differences in rice evolution result in two contrasting types of differentially expressed genes
Source: BMC Genomics. 2015 Dec 23;16:1099. doi: 10.1186/s12864-015-2319-1 (PMC4690246; doi:10.1186/s12864-015-2319-1)
Supplement: Additional file 25: Figure S11. — Relationship between explained variation by detected eQTLs and Zhenshan97 τ. (PDF 455 kb) (PDF 442 kb) [file 12864_2015_2319_MOESM25_ESM.pdf]

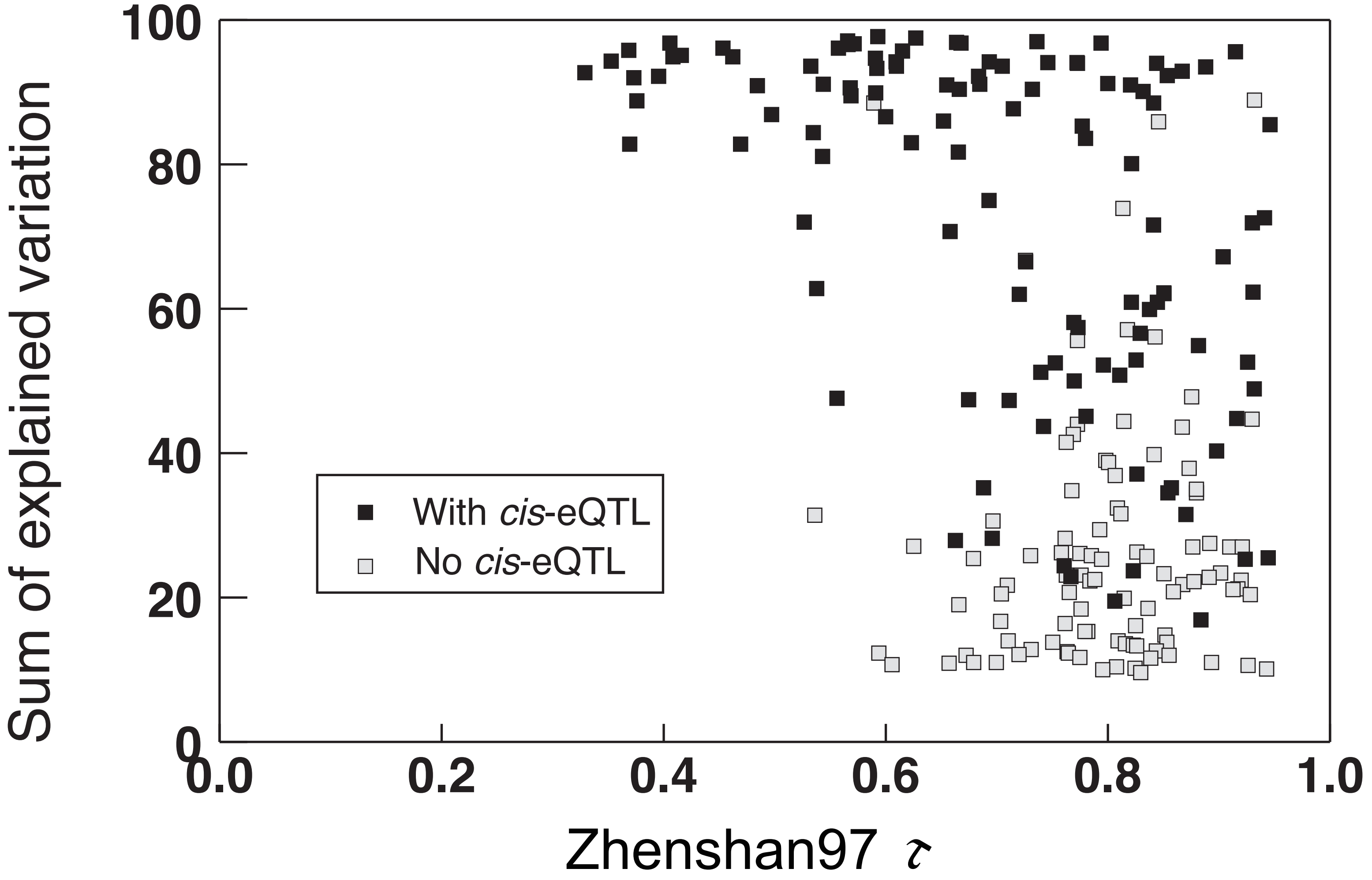

Figure S11. Relationship between explained variation by detected eQTLs and Zhenshan97 *τ*. Scatter plots between Zhenshan97 *τ* and the sum of explained variation by detected eQTLs [20] for changed-tisses MZDE genes at the early seedling stage are shown. The sum of explained variation means a simple summation of explained expression variation across RILs by all detected eQTLs for each gene (Additional file 24: Table S14). Black square genes had *cis*-eQTL among the detected eQTLs. Gray square genes had no *cis*-eQTL among the detected eQTLs.
