# Supplementary material for: Global expression differences and tissue specific expression differences in rice evolution result in two contrasting types of differentially expressed genes
Source: BMC Genomics. 2015 Dec 23;16:1099. doi: 10.1186/s12864-015-2319-1 (PMC4690246; doi:10.1186/s12864-015-2319-1)
Supplement: Additional file 26: Table S15. — Description of the number of unique transcripts and unique probes. (DOCX 111 kb) (DOCX 105 kb) [file 12864_2015_2319_MOESM26_ESM.docx]

**Table S15. Description of the number of unique transcripts and unique probes.**

|  |  | TIGR5^1^ | RAP2^2^ | Affy^3^ | Total |
| --- | --- | --- | --- | --- | --- |
| Original transcripts |  | 56,278 | 53,461 | 57,381 |  |
| Nipponbare unique^4^ | Transcripts | 34,828 | 2,695 | 7,885 | 45,408 |
|  | Probes | 410,802 | 26,162 | 84,473 | 521,437 |
| Nipponbare and 93-11 unique^5^ | Transcripts | 34,555 | 2,656 | 7,824 | 45,035 |
|  | Probes | 401,355 | 25,578 | 83,525 | 510,458 |
| More than two unique probes in a set | Transcripts | 33,786 | 2,411 | 7,737 | 43,934 |
|  | Probes | 400,262 | 25,241 | 83,405 | 508,908 |

^1^ TIGR Rice annotation version 5.0 (ftp://ftp.plantbiology.msu.edu/pub/data/Eukaryotic_Projects/o_sativa/annotation_dbs/pseudomolecules/version_5.0).

^2^ The second Rice Annotation Project (http://rapdblegacy.dna.affrc.go.jp/archive/build4.html).

^3^ Target transcripts used to design rice genome array (http://www.affymetrix.com/Auth/analysis/downloads/data/Rice.consensus.zip). Numbers of unique transcripts and unique probes of RAP2 are shown after eliminations of those redundant with TIGR5. The numbers of Affy were shown after elimination of those redundant with TIGR5 and RAP2.

^4^ Uniquely hit probes on the Nipponbare IRGSPv.4 genome.

^5^ Uniquely hit probes on both the Nipponbare IRGSP v.4 genome sequence and the 93-11 BGI 2003-08-01 genome sequence.
