# Supplementary material for: Global expression differences and tissue specific expression differences in rice evolution result in two contrasting types of differentially expressed genes
Source: BMC Genomics. 2015 Dec 23;16:1099. doi: 10.1186/s12864-015-2319-1 (PMC4690246; doi:10.1186/s12864-015-2319-1)

Figure S2. Dendrogram of 321 arrays from *japonica* Nipponbare (blue), three *indica* varieties 9311 (red), Zhenshan97 (black), and Minghui63 (green). By using 12,097 similarly expressed genes between Nipponbare and 93-11 in both shoot and panicle, 321 arrays were classified into similar tissues. 28 clusters were produced by cutting the dendrogram at height 0.75. Six clusters with red colored branches contain both *japonica* and *indica* array data.

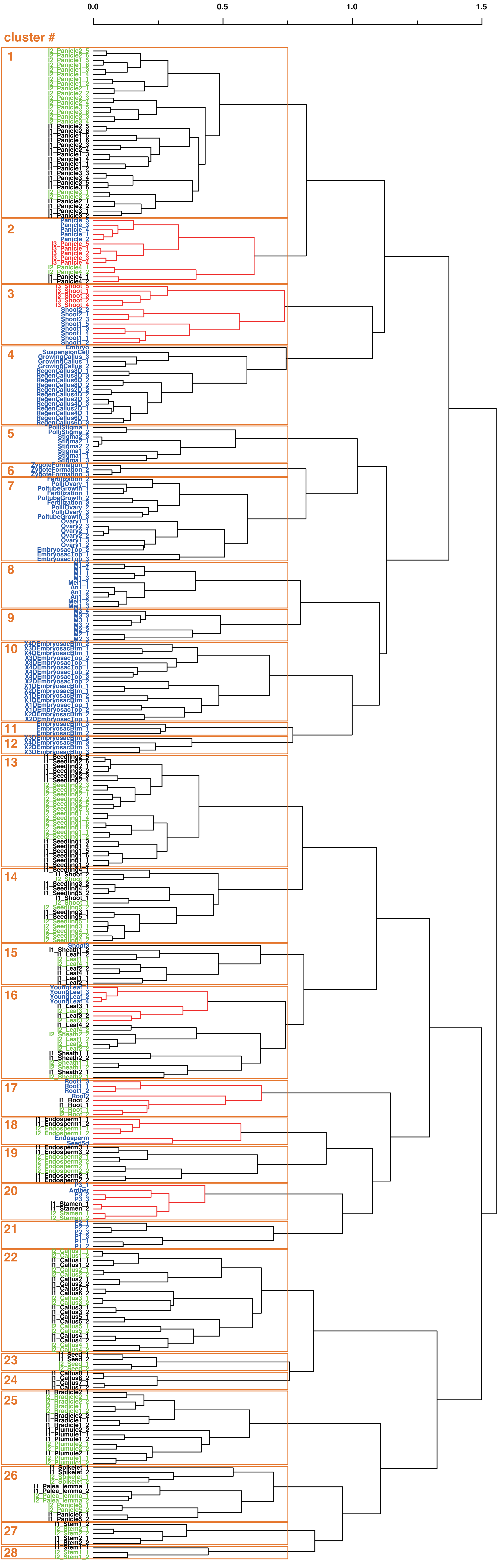

Supplement: Additional file 3: Figure S2. — Dendrogram of 321 arrays from japonica Nipponbare, three indica varieties 93-11, Zhenshan97, and Minghui63. (PDF 340 kb) (PDF 331 kb) [file 12864_2015_2319_MOESM3_ESM.pdf]
